# Next generation marker-based vector concepts for rapid and unambiguous identification of single and double homozygous transgenic organisms

**DOI:** 10.1242/bio.060015

**Published:** 2023-10-19

**Authors:** Frederic Strobl, Julia Ratke, Franziska Krämer, Ana Utta, Sigrun Becker, Ernst H. K. Stelzer

**Affiliations:** Physical Biology / Physikalische Biologie (IZN, FB 15), Buchmann Institute for Molecular Life Sciences (BMLS), Cluster of Excellence Frankfurt – Macromolecular Complexes (CEF – MC), Goethe-Universität Frankfurt am Main (Campus Riedberg), Max-von-Laue-Straße 15, D-60438 Frankfurt am Main, Germany

**Keywords:** Transgenesis, Genotyping, Insect model organisms, *Tribolium castaneum*, Live imaging, Light sheet fluorescence microscopy

## Abstract

For diploid model organisms, the actual transgenesis processes require subsequent periods of transgene management, which are challenging in emerging model organisms due to the lack of suitable methodology. We used the red flour beetle *Tribolium castaneum*, a stored-grain pest, to perform a comprehensive functional evaluation of our AClashOfStrings (ACOS) and the combined AGameOfClones/AClashOfStrings (AGOC/ACOS) vector concepts, which use four clearly distinguishable markers to provide full visual control over up to two independent transgenes. We achieved comprehensive statistical validation of our approach by systematically creating seventeen novel single and double homozygous sublines intended for fluorescence live imaging, including several sublines in which the microtubule cytoskeleton is labeled. During the mating procedures, we genotyped more than 20,000 individuals in less than 80 working hours, which corresponds to about 10 to 15 s per individual. We also confirm the functionality of our combined concept in two double transgene special cases, i.e. integration of both transgenes in close proximity on the same chromosome and integration of one transgene on the X allosome. Finally, we discuss our vector concepts regarding performance, genotyping accuracy, throughput, resource saving potential, fluorescent protein choice, modularity, adaptation to other diploid model organisms and expansion capability.

## Teaser

**Summary:** We present vector concepts that use four phenotypically clearly distinguishable markers to provide full visual control over two independent transgenes, allowing genotyping of double transgenic organisms in less than 15 seconds.

## INTRODUCTION

During the last decades, working with transgenic organisms has become routine in cell and developmental biology (Gama Sosa et al., 2010). When working with diploid model organisms, the actual transgenesis process requires a subsequent period of transgene management, whose scope depends on the properties of the transgene, the transgenesis process, the insertion location, and the scientific question. This period is intended, e.g. for transgene propagation via transmission to subsequent generations, singularization in the case of multiple insertions, creation of homozygous cultures, and/or establishment of hybrid lines. However, depending on the model organism and the respectively available genetic methodology, the practical procedure varies vastly. Transgene management in the fruit fly *Drosophila melanogaster*, the primary insect model organism, has been facilitated by site-specific integration, e.g. by using the ΦC31 integrase system (Venken and Bellen, 2005, 2007), in combination with balancers (Miller et al., 2019). However, for the red flour beetle *Tribolium castaneum*, a stored-grain pest and emerging insect model organism (Brown et al., 2009; Klingler, 2004; Richards et al., 2008), site-specific integration has not yet been established. Hence, transgenesis relies mostly on transposons, e.g. piggyBac (Berghammer et al., 2009; Lorenzen et al., 2003) or Minos (Pavlopoulos et al., 2004), which integrate semi-randomly into the genome. Since only a handful of balancers are known (Denell, 2008; Klingler, 2004; Mocelin and Stuart, 1996), transgene management is more challenging than in *Drosophila*.

In *Tribolium*, a major transgenesis application is the creation of lines suitable for fluorescence live imaging (Strobl and Stelzer, 2016), e.g. for precision functional imaging (Mau et al., 2023). In conjunction with confocal and light sheet fluorescence microscopy (Stelzer et al., 2021; Strobl et al., 2017b), this approach has been used, e.g. to investigate tissue fluidization in the emerging serosa during gastrulation (Jain et al., 2020), to characterize segment formation sequences during germband elongation (Sarrazin et al., 2012), and to observe extra-embryonic membrane dynamics during dorsal closure (Hilbrant et al., 2016). In this context, homozygotization has vast potential to reduce the overall effort and to improve data quality. Firstly, unlike mixed cultures, homozygous cultures do not require constant curation, which simplifies stock keeping. Secondly, descendants of homozygous parents are always homozygous, while a culture with mixed genotypes produces a combination of wild types, hemizygotes and homozygotes. Only the latter two are suited for fluorescence microscopy, and arbitrary choice introduces an adverse nuisance factor to the experiment. Thirdly, studies involving transgenic organisms of other species indicate that homozygous individuals provide a stronger fluorescence signal than their hemizygous relatives (Cornett et al., 2011; Lin et al., 2015).

Transgene management in *Tribolium* is still cumbersome, mainly since conventional non-lethal genotyping approaches, e.g. test crosses and/or genetic assays, are labor- as well as resource-intensive and only available for adult individuals (Strobl et al., 2017c). However, the improvement of transgene management for diploid model organisms, including *Tribolium*, established with AGameOfClones (AGOC), the AClashOfStrings (ACOS), and the combined AGOC/ACOS vector concepts (Strobl and Stelzer, 2021; Strobl et al., 2018), which are based on four phenotypically distinguishable markers, provide full visual control over up to two independent transgenes. In this study, we performed an extended functional evaluation of our approach. Firstly, we achieved a comprehensive statistical validation of our concepts by genotyping more than 20,000 individuals and confirming the results in more than 160 cases by scoring the progeny, thereby creating ten novel homozygous ACOS sublines and seven novel double homozygous AGOC/ACOS hybrid sublines. Secondly, we assayed the functionality of AGOC/ACOS in double transgene special cases where either (i) both transgenes are located on the same autosome or (ii) one transgene is located on the X allosome. Thirdly, we assayed transgenesis-based strategies for cell membrane and microtubule cytoskeleton labeling in *Tribolium*. Since our vectors are primarily designed to create fluorescence reporter lines, we also performed live imaging assays of early embryogenesis, i.e. of the last three synchronous division waves during blastoderm formation, using selected ACOS sublines and AGOC/ACOS hybrid sublines. Finally, we discuss our vector concepts regarding aspects such as performance, genotyping accuracy, throughput, resource saving potential, fluorescent protein choice, modularity, transgene management in transgenesis special cases, adaptation to other diploid model organisms, ethical considerations and expansion capabilities.

## RESULTS

### Vector architecture and establishment of ACOS lines/sublines

Similarly to our two previous studies, the extended functional evaluation of ACOS and AGOC/ACOS relied on *Tribolium* as well as on the piggyBac transposon system (Berghammer et al., 2009; Lorenzen et al., 2003), which inserts transgenes semi-randomly, i.e. targeting TTAA sites, into the genomes of the respective organisms. At first, we created the pACOS{#P′#O(MEM)-mRuby} vector (Figs S1 and S2) that contains mCerulean-based (Markwardt et al., 2011) and mVenus-based (Nagai et al., 2002) eye-specific (Berghammer et al., 1999) transformation markers (mCe and mVe, respectively). These markers are visually clearly distinguishable from each other as well as from those of AGOC, which are based on mOrange and mCherry. Since both markers are placed within interweaved LoxP/LoxN site pairs, Cre-mediated recombination excises either mCe or mVe. Accordingly, the respective other marker remains within the transgene as the remaining single LoxP and LoxN sites are incompatible with each other (Fig. S3A). Based on this vector, we created two derivates in which the mRuby2-labeled (Lam et al., 2012) GAP43 membrane anchor tag (MEM), which has already been used in *Tribolium* mRNA injection-based labeling assays (Benton et al., 2013), is expressed under control of either the *tubulin alpha 1-like protein* promoter (ATub′) (Siebert et al., 2008) or the *polyubiquitin* promoter (Ub′) (Lorenzen et al., 2002). Furthermore, we created three additional derivates in which mRuby2-labeled *tubulin alpha 1-like protein* (ATub) is expressed under control of either the aforementioned promoters or a shortened version of the *elongation factor 1-alpha* promoter (ShEFA′) (Peel and Averof, 2010; Sarrazin et al., 2012).

We injected these transformation-ready vectors together with pATub′piggyBac (Strobl et al., 2018), a piggyBac transposase-expressing helper vector, into pre-blastoderm embryos to achieve germline transformation. We mated all survivors, i.e. F1 potential mosaics, with wild types, and found in 17 of these crosses one or more potential F2 (mCe-mVe) founder females among the descendants. At first, we estimated the insert number by mating each of these females with one wild-type male and scoring the progeny. To establish respective sublines (Table S1, first and second column), we used the pair with the lowest ratio of transgenic descendants (Table S1, third column) to ensure that only one insert is present. Next, we estimated homozygous viability by mating two F3 (mO-mC) pre-recombination hemizygous siblings and scoring the progeny, which indicated that out of the 17 sublines, only nine were assumed to be homozygous viable (Table S1, fourth column). Additionally, we performed inverse PCR-based insertion junction sequencing, which was successful for 11 of the sublines, including eight of the nine assumingly homozygous viable ones (Table S2).

### The ACOS vector concept: systematic creation of homozygous transgenic sublines

The primary application of ACOS is the systematic creation of homozygous transgenic sublines. The associated mating procedure runs from the F3 to the F7 generation (Fig. S3B) and involves a transgenic helper subline, FIRE{HSP68′NLS-Cre} #1, which carries a transgene on the X allosome that mediates expression of a nuclear-localized Cre recombinase (Peitz et al., 2002) under control of the *heat shock protein 68b* promoter (Schinko et al., 2012) and is accompanied by mC:

1. F3 (mCe-mVe) pre-recombination hemizygous females, which carry mCe and mVe on the maternal chromosome in cis configuration, are mated with (mC) helper males (Fig. S3B, ‘F3’ rectangles). This results in F4 (mC; mCe-mVe) double hemizygotes, in which Cre-mediated recombination occurs. In this hybrid generation, adults usually display a patchy expression of mCe and mVe within their compound eyes (Fig. S4).
2. F4 (mC; mCe-mVe) double hemizygous females are mated with wild-type males (Fig. S3B, ‘F4’ rectangles). Due to Cre-mediated recombination in the germline, this results in F5 (mCe) and (mVe) post-recombination hemizygotes that carry either only mCe or only mVe on the maternal chromosome.
3. F5 (mCe) post-recombination hemizygous females are mated with F5 (mVe) post-recombination hemizygous male siblings (Fig. S3B, ‘F5’ rectangles), which results in F6 (mCe/mVe) heterozygotes that carry mCe on the maternal and mVe on the paternal chromosome in trans configuration.
4. F6 (mCe/mVe) heterozygous females are mated with genotypically identical male siblings (Fig. S3B, ‘F6’ rectangles). This results in F7 (mCe/mCe) and (mVe/mVe) homozygotes that carry either only mCe or only mVe on both the maternal and paternal chromosome (Fig. S3B, ‘F7’ rectangles), which are used to establish F7+ continuative cultures.

**Fig. 1.**
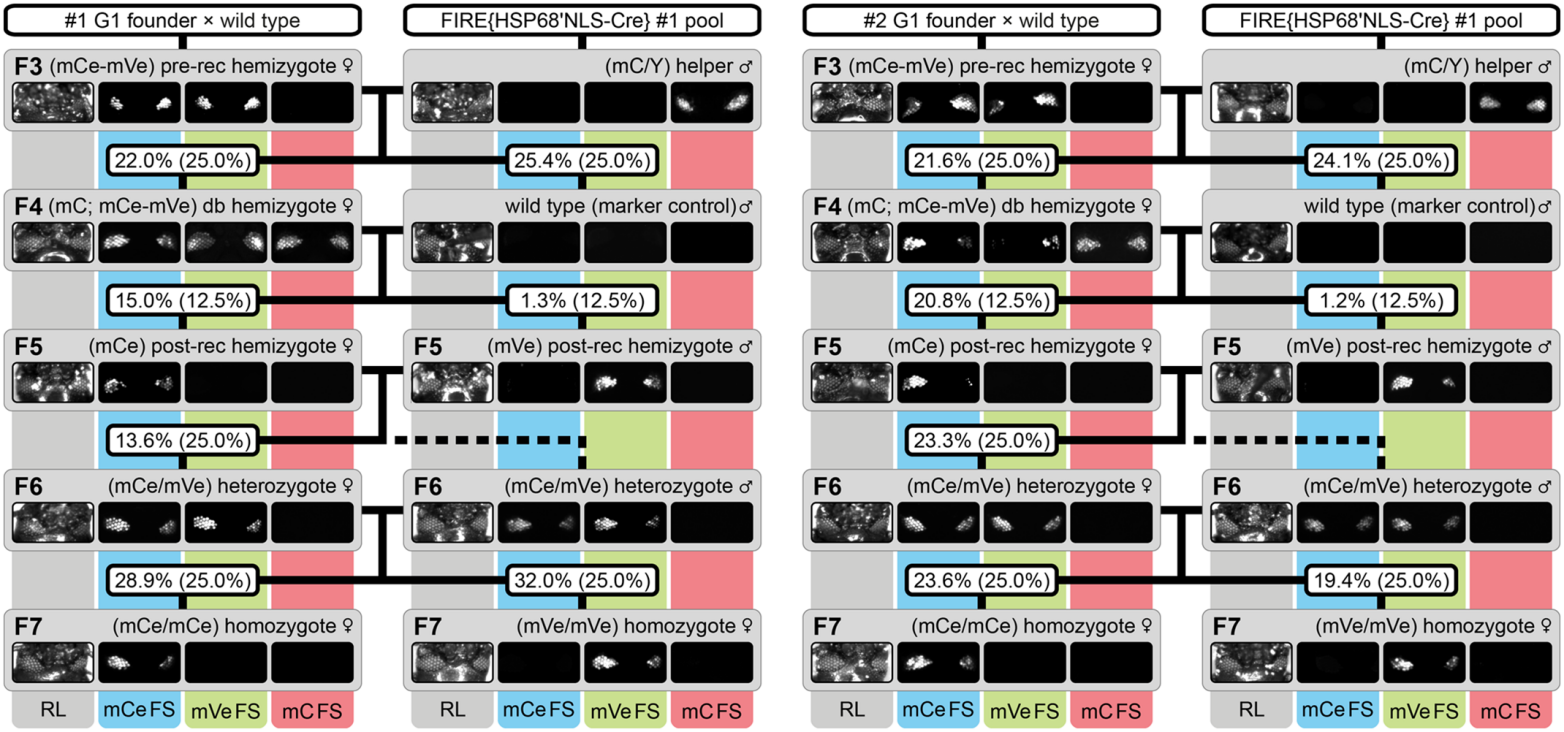
The ACOS-associated mating procedure from the F3 to the F7 generation demonstrated for the ACOS{ATub′#O(MEM)-mRuby #1 and #2 sublines. Genotypes were phenotypically determined by monitoring mC, mCe and mVe. Eventually, F7 (mCe/mCe) and (mVe/mVe) homozygotes were obtained for both sublines by following the mating procedure outlined in Fig. S3B. The respective wild-type males to the right of the ‘F4’ rectangles function as marker controls. The percentage boxes indicate the experimental (and theoretical) ratios of the progeny that displayed the respective phenotype, the dashed lines represent genotypically identical siblings. FS, filter set; rec, recombination; db, double.

We commenced the procedure with all seventeen sublines and were able to obtain F6 heterozygotes as well as both ‘flavors’ of F7 homozygotes for ten of them, as the respective control crosses demonstrate (Table S3). Surprisingly, of the nine sublines initially assumed to be homozygous viable, only eight went through the procedure successfully. This included the ACOS{ATub′#O(MEM)-mRuby} #1 and #2 sublines, for which we documented the markers throughout the generations (Fig. 1). More surprisingly, of the eight sublines that were assumed to be homozygous lethal, two went through the procedure successfully, leaving a total of seven sublines for which the mating procedure aborted due to a lack of suitable progeny. This included all ACOS{Ub′#O(MEM)-mRuby} sublines (Table S4).

In the F3, F5, F6 (Table 1), F6-S, F6-mCe, and F7-mVe crosses (Table S2), the arithmetic means of the progeny ratios from all ten homozygous viable sublines did not deviate significantly from the theoretical ratios, which are solely based on the Mendelian inheritance principles. In the F4 cross, the situation is more complex since the theoretical ratios are based on a combination of Mendelian distribution and Cre-associated recombination stochastics. Previous studies suggest that two interweaved Lox site pairs, as present in our vector concept, recombine with almost equal probability if the sequence length between the Lox site pairs is similar (Wang et al., 2009). Hence, we hypothesized that the theoretical 25% of the progeny that would inherit only the ACOS transgene evenly split up into twice 12.5% that carry either only mCe or only mVe. Consequently, we assumed that this also applies to the theoretical 25% of the progeny that would inherit, besides the ACOS transgene, also the helper transgene that is accompanied by mC. Unexpectedly, the Cre recombinase significantly favored the LoxP sites and excised the mVenus-based eye-specific transformation marker about five times more often. However, since the F4 crosses always resulted in sufficient (mVe) post-recombination hemizygotes, the mating procedure could be continued without restrictions.

**Table 1.**
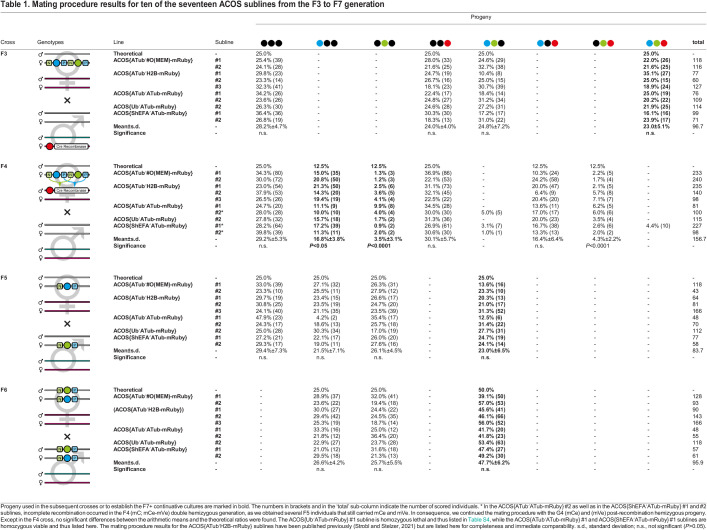
Mating procedure results for ten of the seventeen ACOS sublines from the F3 to F7 generation

**Fig. 2.**
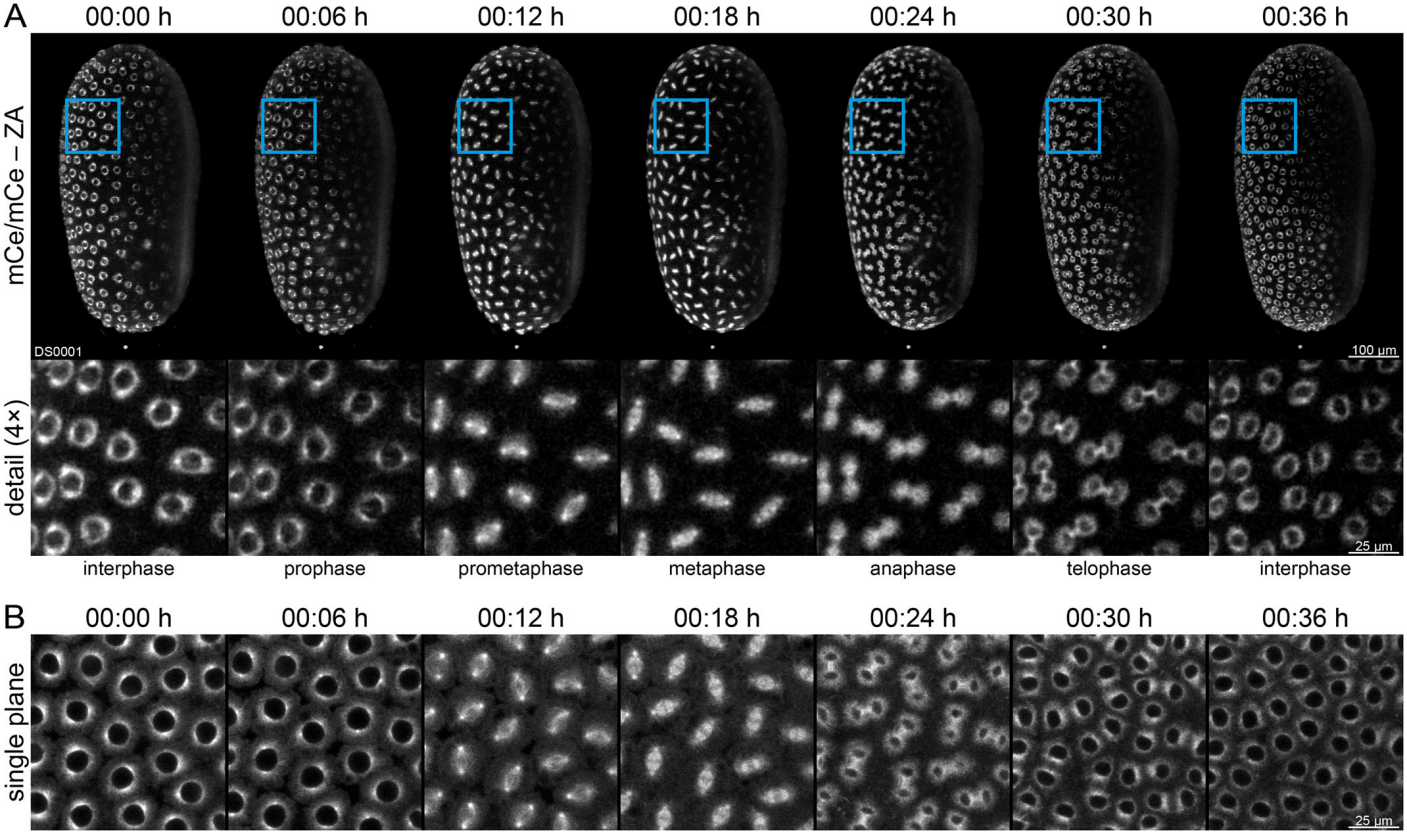
**Fluorescence live imaging of homozygous ACOS{Ub**′**ATub-mRuby} #2 embryos during blastoderm formation.** This subline expresses mRuby2-labeled *tubulin alpha 1-like protein* under control of the *polyubiquitin* promoter. (A) *In toto* time series of a (mCe/mCe) embryo proceeding through the 10th synchronous division wave using light sheet fluorescence microscopy. The intracellular label allows identification of mitotic phases. ZA, Z maximum projection with intensity adjustment. (B) High magnification single-plane time series of a (mCe/mCe) embryo proceeding through the 10th synchronous division wave using confocal laser scanning microscopy. Clearly visible are the excellent resolution, good signal-to-noise ratio, and high dynamic range, hence cytosolic components are easily identified.

To evaluate the suitability of the ACOS sublines for fluorescence microscopy, we imaged (mCe/mCe) homozygous embryos from the ACOS{ATub′#O(MEM)-mRuby} #1 and #2 (Fig. S5A) and (mCe) hemizygous embryos from the ACOS{Ub′#O(MEM)-mRuby} #1 and #2 sublines (Fig. S5B and C). The data indicate that the GAP43 membrane anchor tag (MEM) also principally functions in *Tribolium* when used in transgenesis-based fluorescence labeling approaches. However, signal from the *tubulin alpha 1-like protein* promoter-based expression cassettes was too faint, while fluorescence signal in the *polyubiquitin* promoter-based sublines was found almost exclusively within the serosa and appears too patchy for adequate characterization of membrane dynamics via live imaging. Further, we imaged (mCe/mCe) homozygous ACOS{Ub′ATub-mRuby} #2 embryos during blastoderm formation with more success (Fig. 2). This subline allows identification of the successive mitotic phases, e.g. during the 10th synchronous division wave of blastoderm formation. Consequently, we compared imaging data from (mCe/mCe) and (mVe/mVe) homozygous embryos, which indicated that there is no apparent difference between both flavors (Fig. S6, Movie 1).

### The combined AGOC/ACOS vector concept: systematic creation of double homozygous transgenic hybrid sublines

Both AGOC and ACOS were initially designed with complementarity in mind, i.e. AGOC/ACOS can be used for the systematic creation of double homozygous transgenic sublines. The associated mating procedure runs from the F7 to the F10 generation (Fig. S7, a comprehensive scheme is provided in Fig. S8):

1. F7 (mO/mO) homozygous females (AGOC), which carry only mO on both parental chromosomes, are mated with F7 (mCe/mCe) homozygous males (ACOS), which carry only mCe on both parental chromosomes (Fig. S7, ‘F7’ rectangles on the left). In parallel, F7 (mC/mC) homozygous females (AGOC), which carry only mC on both parental chromosomes, are mated with F7 (mVe/mVe) homozygous males (ACOS), which carry only mVe on both parental chromosomes (Fig. S7, ‘F7’ rectangles on the right). This results in F8 (mO; mCe) and (mC; mVe) double hemizygotes.
2. F8 (mO; mCe) double hemizygous females are mated with F8 (mC; mVe) double hemizygous males (Fig. S7, ‘F8’ rectangles), which results in F9 (mO/mC; mCe/mVe) double heterozygotes that carry all four markers.
3. F9 (mO/mC; mCe/mVe) double heterozygous females are mated with genotypically identical F9 male siblings (Fig. S7, ‘F9’ rectangles). This results in F10 (mO/mO; mCe/mCe), (mC/mC; mCe/mCe), (mO/mO; mVe/mVe) and (mC/mC; mVe/mVe) double homozygotes (Fig. S7, ‘F10’ rectangles), which are used to establish F10+ continuative cultures.

**Fig. 3.**
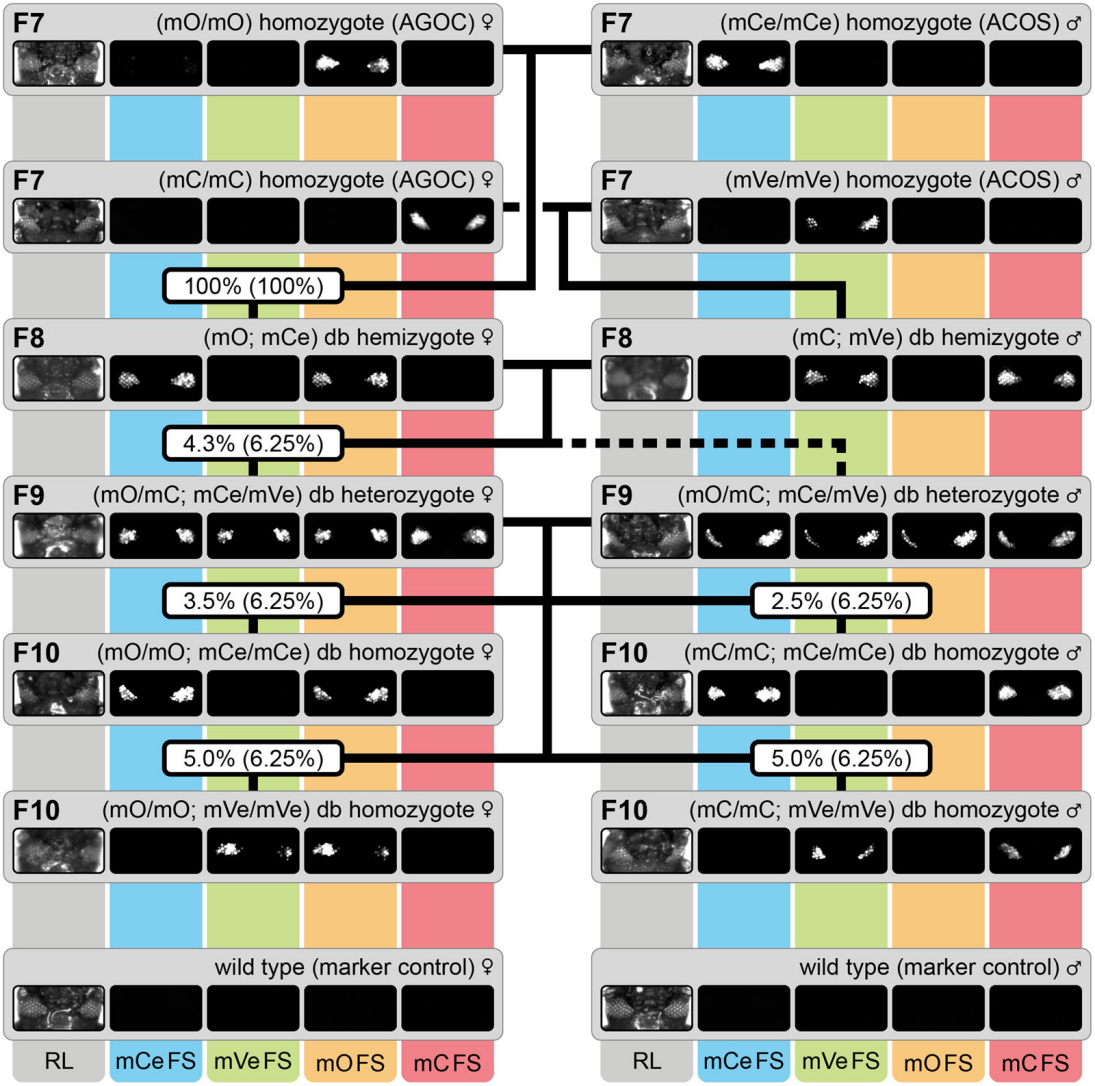
**The AGOC/ACOS-associated mating procedure from the F7 to the F10 generation demonstrated for the Gruul #4 subline.** Genotypes were phenotypically determined by monitoring mCe, mVe, mO and mC. Eventually, F10 (mO/mO; mCe/mCe), (mC/mC; mCe/mCe), (mO/mO; mVe/mVe) and (mC/mC; mVe/mVe) double homozygotes were obtained by following the mating procedure outlined in Fig. S7. The respective wild types below the ‘F10’ rectangles function as marker controls. The percentage boxes indicate the experimental (and theoretical) ratios of the progeny that displayed the respective phenotype, the dashed line represents genotypically identical siblings. FS, filter set; rec, recombination; db, double.

With regard to the results from the insertion junction sequencing assays (Table S2), we commenced the AGOC/ACOS-associated mating procedure with five AGOC/ACOS subline pairs that carry their two respective transgenes on different chromosomes (Table S5, fourth to eighth row). In accordance with our previous study (Strobl and Stelzer, 2021), we named these hybrid sublines Gruul #4 to #8. All five sublines, including Gruul #4, for which we documented the markers throughout the generations (Fig. 3), went through the procedure successfully (Table 2). Consequently, we obtained F9 double heterozygotes and all four flavors of F10 double homozygotes for each subline, as the respective control crosses demonstrate (Table S6).

**Table 2.**
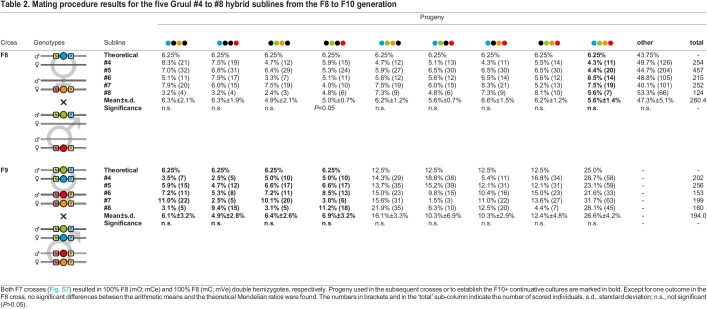
Mating procedure results for the five Gruul #4 to #8 hybrid sublines from the F8 to F10 generation

Throughout the AGOC/ACOS-associated mating procedure, we only experienced one instance in which the arithmetic mean of the progeny ratio significantly differed from the respective theoretical ratio. The F8 crosses resulted, as expected, in progeny with sixteen different genotypes, but the mean ratio of (mC; mCe) double hemizygotes (5.0%±0.7%) was significantly lower than the expectations (6.25%, *P*<0.05). However, since the number of F9 (mO/mC; mCe/mVe) double heterozygotes matched the expectations and were sufficient, the mating procedure could be continued as intended.

### The combined AGOC/ACOS vector concept in double transgene special cases

So far, our results showed that AGOC/ACOS can be used for the systematic creation of double homozygous lines in cases where both transgenes are located on different autosomes. We tested the applicability of our concept in two double transgene special cases: (i) both transgenes are located in close proximity on the same autosome and (ii) one transgene is located on an autosome while the other is located on the X allosome.

If both transgenes are located on the same autosome, the mating procedure does not change. However, deviations from theoretical Mendelian ratios are expected since multiple meiotic recombination events in the region between both transgenes are required:

1. When F8 (mO; mCe) double hemizygous females are mated with F8 (mC; mVe) double hemizygous males, both maternal and paternal meiotic recombination are required to obtain F9 (mO/mC; mCe/mVe) double heterozygotes.
2. When F9 (mO/mC; mCe/mVe) double heterozygous females are mated with genotypically identical F9 male siblings, both maternal and paternal meiotic recombination are again required to obtain F10 (mC/mC; mCe/mCe) and (mC/mC; mVe/mVe) homozygotes.

Our insertion junction sequencing assays (Table S2) revealed that both the ACOS{ATub′#O(MEM)-mRuby} #2 and the AGOC{ATub′H2B-mEmerald} #3 sublines carry their transgenes on ChLG9, in the TTAA sites at positions 8,613,308-11 and 10,781,551-4, respectively, i.e. with a chromosomal distance of about 2.17 Mbp, and were therefore a convenient match to investigate the first special case. We named this hybrid subline Gruul #9 and commenced the mating procedure in three independent repetitions, which all went through the procedure successfully (Table 3). Consequently, we obtained F9 double heterozygotes as well as all four flavors of F10 double homozygotes for each repetition, as the respective control crosses demonstrate (Table S7). As expected, the arithmetic means of the progeny ratios differed from the respective theoretical Mendelian ratios in all 23 assayed instances. For the eight genotypes for which no meiotic recombination was necessary, the mean was significantly higher (5× *P*<0.01, 3× *P*<0.05), and for genotypes for which either maternal or paternal as well as maternal and paternal meiotic recombination were necessary, the mean was significantly lower (2× *P*<0.001, 5× *P*<0.01, 3× *P*<0.05 and 1× *P*<0.0001, 3× *P*<0.001, 1× *P*<0.01, respectively).

**Table 3.**
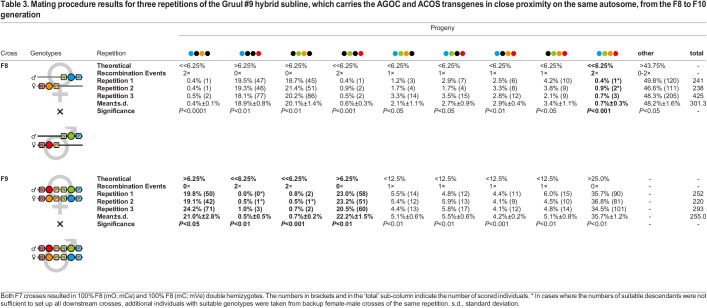
Mating procedure results for three repetitions of the Gruul #9 hybrid subline, which carries the AGOC and ACOS transgenes in close proximity on the same autosome, from the F8 to F10 generation

The progeny scoring results from the F9, F9 and F9-S crosses were further used to calculate the genetic linkage between two transgenes that inserted on the same chromosome. In the three Gruul #9 hybrid subline mating procedure run repetitions, for a total of 1,248; 1,171; and 1,651 gametes, 98, 96, and 147 occurrences of meiotic recombination in between both transgenes were scored, which translates to a genetic linkage of 8.31 cM±0.54 cM.

Further, if one transgene is located on an autosome and the other is located on the X allosome, the mating procedure becomes slightly more complex (Fig. S9):

1. F7 (mO/mO) homozygous females (AGOC), which carry only mO on both X allosomes, are mated with F7 (mCe/mCe) homozygous males (ACOS), which carry only mCe on both parental autosomes (Fig. S9, ‘F7’ rectangles on the left). In parallel, F7 (mC/mC) homozygous females (AGOC), which carry only mC on both X allosomes, are mated with F7 (mVe/mVe) homozygous males (ACOS), which carry only mVe on both parental autosomes (Fig. S9, ‘F7’ rectangles on the right). This results in F8 (mO; mCe) and (mC; mVe) double hemizygotes.
2. F8 (mO; mCe) double hemizygous females are mated with F8 (mC/Y; mVe) double hemizygous males (Fig. S9, ‘F8’ rectangles on the left), which results in F9 (mO/mC; mCe/mVe) double heterozygous females and (mO/Y; mCe/mVe) hemi-heterozygous males. In parallel, F8 (mC; mVe) double hemizygous females are mated with F8 (mO/Y; mCe) double hemizygous males (Fig. S9, ‘F8’ rectangles on the right), which results in F9 (mCe/mVe; mO/mC) double heterozygous females, which are genotypically identical to the females that result from the other F8 cross, and (mC/Y; mCe/mVe) hemi-heterozygous males.
3. F9 (mO/mC; mCe/mVe) double heterozygous females are mated with (mO/Y; mCe/mVe) hemi-heterozygous males (Fig. S9, ‘F9’ rectangles on the left) and, in parallel, with (mC/Y; mCe/mVe) hemi-heterozygous males (Fig. S9, ‘F9’ rectangles on the right). This results in all four flavors of F10 double homozygous females and the corresponding (mO/Y; mCe/mCe), (mC/Y; mCe/mCe), (mO/Y; mVe/mVe) and (mC/Y; mVe/mVe) hemi-homozygous males (Fig. S9, ‘F10’ rectangles) that are used to establish F10+ continuative cultures.

Our insertion junction sequencing assays (Table S2) revealed that AGOC{Zen1′#O(LA)-mEmerald} #2 carries the transgene on ChLGX, while ACOS{ATub′H2B-mRuby} #1 carries the transgene on ChLG6. Hence, both sublines were a convenient match to investigate the second special case. We named this hybrid subline Gruul #10 and commenced the mating procedure in three independent repetitions, which all went through the procedure successfully (Table 4). Consequently, we obtained F9 double heterozygous females as well as all four flavors of F10 double homozygous females and respective males for each repetition, as the respective control crosses demonstrate (Table S8). As expected, the arithmetic means of the progeny ratios did not differ from the respective theoretical Mendelian ratios in all 23 assayed instances.

**Table 4.**
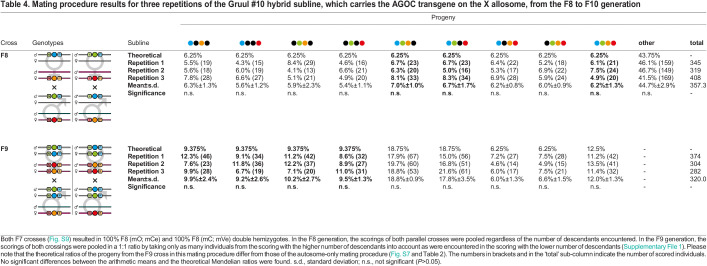
Mating procedure results for three repetitions of the Gruul #10 hybrid subline, which carries the AGOC transgene on the X allosome, from the F8 to F10 generation

### The AGOC/ACOS vector concept for the creation of double transgenic fluorescence reporter lines

**Fig. 4.**
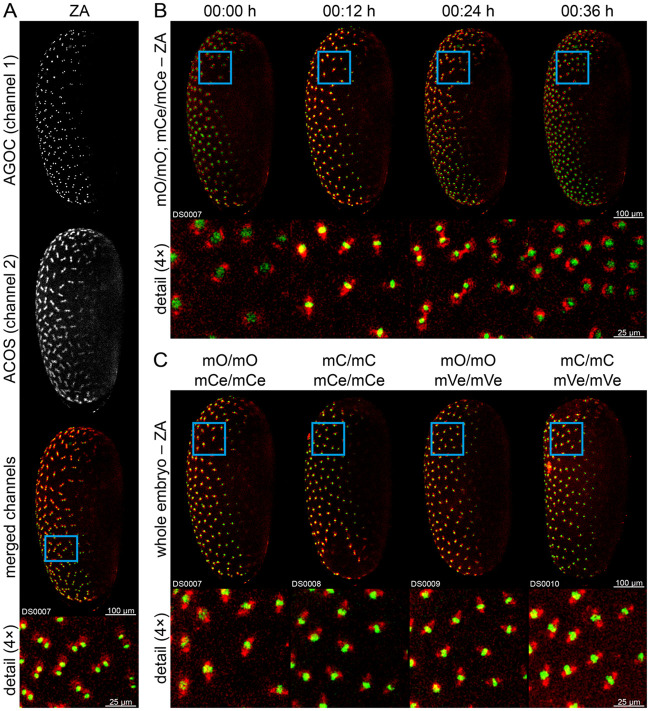
**Fluorescence live imaging of double homozygous Gruul #8 embryos during blastoderm formation in two channels using LSFM.** This hybrid subline expresses mEmerald-labeled *histone H2B* under control of the *tubulin alpha 1-like protein* promoter and mRuby2-labeled *tubulin alpha 1-like protein* under control of a shortened version of the *elongation factor 1-alpha* promoter. (A) Fluorescence channel juxtaposition of a (mO/mO; mCe/mCe) embryo. (B) Time series of a (mO/mO; mCe/mCe) embryo proceeding through the 10th synchronous division wave. (C) Comparison of differently flavored hybrid sublines. All embryos show similar fluorescence patterns. ZA, Z maximum projection with intensity adjustment.

To evaluate the suitability of the AGOC/ACOS hybrid sublines for fluorescence microscopy, we imaged (mO/mO; mCe/mCe) double homozygous Gruul #8 embryos during blastoderm formation in two channels (Fig. 4A). This hybrid subline allows observation of chromatin condensation and spindle apparatus dynamics during mitosis, e.g. during the 10th synchronous division wave of blastoderm formation (Fig. 4B). Furthermore, we compared imaging data from all four flavors of the F10 double homozygotes, which indicated no apparent differences in the nature of the signal (Fig. 4C). All datasets show the 10th, 11th, and 12th synchronous division waves, i.e. the last three waves that occur on the surface of the yolk before the blastoderm has fully formed. However, while the mEmerald-derived signal persists throughout blastoderm formation, the mRuby-derived signal gradually fades, which hinders identification of the spindle apparatus during the 12th wave (Movie 2).

## DISCUSSION

In this study, we performed a comprehensive statistical verification of ACOS and AGOC/ACOS for high-throughput genotyping and the systematic creation of single homozygous lines as well as double homozygous hybrid sublines and confirmed the functionality of AGOC/ACOS experimentally in two double-transgene special cases. We also established microtubule labeling for *Tribolium* and used respective embryos in conjunction with light sheet fluorescence microscopy to collect live imaging data of the last three synchronous division waves during blastoderm formation. Taken together, our vector concepts perform as intended and facilitate transgene management considerably, but the not so obvious but extremely interesting peculiarities will be discussed and explained in more detail.

### Homozygous viability of ACOS-based transgenic sublines

Especially for transgenic fluorescence reporter lines, working with sublines homozygous for the transgene is preferable, as it eliminates the need for constant curation, removes a nuisance factor, and probably provides a stronger fluorescence signal. Therefore, it is reasonable to assess homozygous viability of newly created sublines as early as possible. Throughout the experimental part of our study, we created a total of seventeen ACOS-based sublines, for which the ACOS-associated mating procedure demonstrated in the F6 generation indubitably that ten are homozygous viable. When these results are compared with the conclusions from the homozygous viability estimation assay (Table S4), it becomes evident that the latter gave false-negative indications for the ACOS{ATub′ATub-mRuby} #2 and ACOS{ShEFA′ATub-mRuby} #1 sublines and a false-positive indication for the ACOS{Ub′ATub-mRuby} #1 subline. This indicates that preliminary homozygous viability estimation assays may occasionally provide unreliable suggestions and that our vector concepts are well-suited for proper validation or could even obviate the need for those assays.

Further, the seven homozygous lethal sublines include all six ACOS{Ub′#O(MEM)-mRuby} sublines. Since insertion junction sequencing was not successful for any of these sublines, we could not determine whether the respective transgenes impair endogenous genes. However, a large-scale insertional mutagenesis screening study in *Tribolium* (Trauner et al., 2009) indicates that such an ‘indirect’ recessive lethality occurs only in about 7% of all insertions. In turn, this suggests that the respective transgene, if present in two doses, causes lethality directly and systematically. This assumption is also supported by the fact that we found four F6 (mCe/mVe) larvae during the #6 subline mating procedure run, which, however, had a rather lethargic demeanor and deceased before pupation (Table S4). So which attributes of the transgene could lead to homozygous lethality? Our vector architecture was, in principle, inspired by pCS2-GAP43-YFP (Montero et al., 2005). This vector is primarily used for *in vitro* transcription of injection-ready mRNA encoding the YFP-labeled human GAP43 membrane anchor tag, and a previous study has successfully verified the functionality of this approach in *Tribolium* (Benton et al., 2013). However, there are two crucial differences to our approach. Firstly, mRNA-based labeling is transient, and the respective individuals do not have to cope with an artificial genetic modification for their entire existence. Secondly, our linker sequence between the anchor tag and mRuby2 is, with only three amino acids, relatively short. Hence, it is possible that the fusion protein sterically hampers interaction between membrane and cytosol, especially at high densities, as expected when expression is mediated by the *polyubiquitin* promoter (Lorenzen et al., 2002). In contrast, the two ACOS{ATub′#O(MEM)-mRuby} sublines are homozygous viable, but this may be due to the fact that the *tubulin alpha 1-like protein* promoter (Siebert et al., 2008) is assumed to be considerably weaker than the *polyubiquitin* promoter (Lorenzen et al., 2002).

Regarding the two ACOS{Ub′ATub-mRuby} sublines, #1 turned out to be homozygous lethal. Insertion junction sequencing revealed that the transgene integrated between the first and second exon of the *4-hydroxybutyrate coenzyme A transferase-like protein* gene (TC034329). Since exon 1 contains the only putative transcription initiation site of this gene, the transgene possibly impairs gene function entirely. In contrast, the #2 subline is homozygous viable without noticeable restrictions and provides convenient signal for live imaging. Hence, a high expression level of mRuby2-labeled *tubulin alpha 1-like protein* does not systematically lead to homozygous lethality. Taken together, these results suggest that our current combination of the *polyubiquitin* promoter and membrane labeling approach is suboptimal, and we will continue experimentation to create ACOS-based membrane-labeled sublines that will, in conjunction with AGOC, enable the systematic creation of diverse combinations of two-colored double homozygous hybrid sublines.

### Genotyping accuracy, throughput, and resource saving potential

Regarding genotyping accuracy, our concepts showed flawless performance throughout all successfully completed single and double transgene mating procedure runs, as the phenotypically determined genotypes of parents and their progeny harmonized in all 56 ACOS- and all 110 AGOC/ACOS-related scorings (Supplementary File 1). This suggests that both concepts are as reliable as AGOC and, therefore, altogether a convenient approach to avoid confusion, severe backlashes and/or false conclusions in workflows that involve large numbers of transgenic individuals.

ACOS and AGOC/ACOS are, similar to AGOC (Strobl et al., 2018), designed for high-throughput genotyping and, consequently, to reduce working time and save resources. Throughout the experimental phase of our study, we assayed 5,929 individuals during the ACOS mating procedure runs and 14,214 individuals during the AGOC/ACOS mating procedure runs, which sums up to 20,143 total genotypings (Supplementary File 1). Suppose the average time required to assay one individual, including filter set change and documentation of results, is about 10 and 15 s, respectively, the total working time equals less than 80 h. If the same number should be genotyped with genetic assays, for example by using a protocol based on genomic DNA extraction from dissected wing tissue (Strobl et al., 2017c), the required working time is one to two orders of magnitude longer.

Further, continuous mating of transgenic organisms is subject to stochastic principles. Consequently, the production of a certain number of individuals with suitable genotypes is always accompanied by significant additional effort. In single transgene mating schemes, in the worst case, only 25% of the progeny carry the desired genotype, e.g. if homozygous transgenic descendants should be obtained by mating two heterozygous siblings (cf. Fig. S3). In double transgene mating schemes, the situation is even more pessimistic. In the worst case, only 6.25% of the progeny carry the desired genotype, e.g. if double homozygous transgenic descendants should be obtained by mating of two double heterozygous siblings (Fig. S7). In *Tribolium*, the genotype cannot be immediately assayed as test crosses and, e.g. the aforementioned wing tissue-based protocol are restricted to adults. This means that large numbers of individuals need to be maintained over long periods of time before a suitable fraction can be identified (Strobl and Stelzer, 2021), which is about 3 weeks for *Tribolium*. In contrast, our vector concepts allow genotyping directly after nascence, which allows to discontinue progeny production immediately after the necessary number of suitable individuals is reached. In the case of *Tribolium*, this saves flour, multi-well plates, and space within the incubator. Adaptions of our concept for other diploid model organisms will result in similar resource savings.

### Performance of mRuby2 in *Tribolium*

Throughout our live imaging assays, mRuby2 (Lam et al., 2012), our fluorescent protein of choice for microscopic analyses, showed only mediocre overall performance in *Tribolium*. As shown previously, if mRuby2 accumulates within the cell, e.g. within the nucleus as in the ACOS{ATub′H2B-mRuby} sublines, sufficient signal can be obtained even over long imaging periods (Strobl and Stelzer, 2021). However, in *Tribolium*, this fluorescent protein appears inconvenient to label more delicate subcellular structures. The signal of both ACOS{ATub′#O(MEM)-mRuby} sublines is so faint that live imaging is basically impossible, all ACOS{Ub′#O(MEM)-mRuby} sublines provide patchy signal, and all sublines expressing mRuby2-labeled *tubulin alpha 1-like protein*, independently of the promoter, provide convenient signal only during blastoderm formation. We decided for mRuby2 since its 559 nm excitation peak is close to the 561 nm laser line, which is a given standard in confocal and light sheet fluorescence microscopes, and since it has one of the highest quantum yields of all red fluorescent proteins that were known at the time when the choice was made. Alternatives with excitation peaks similarly close to 561 nm, which we consider to evaluate in the future, are tdTomato (Shaner et al., 2004), mNectarine (Johnson et al., 2009), and RRvT (Wiens et al., 2016). Although mCherry is a common choice for fluorescence live imaging in the red regime (Shaner et al., 2005), we tend to avoid it in relation to our concepts, as it is already part of one of the AGOC markers.

### Live imaging of early embryogenesis

In the live imaging part of our study, we focused on blastoderm formation, i.e. the very early embryogenesis, which was non-trivial since *Tribolium* embryos are rather fragile during this developmental stage. In order to ensure viability, we had to halve the incubation time in sodium hypochlorite solution, a necessary pre-imaging procedure for removing the autofluorescent chorion from the embryos. In consequence, small to medium chorion fragments occasionally remained on top of the vitelline membrane, e.g. in DS0003, DS0004 and DS0006 (Movie 1). While single-channel live imaging of embryos from the ACOS{Ub′ATub-mRuby} #2 subline was straightforward, two-channel imaging of embryos from the Gruul #8 hybrid subline remained challenging. To obtain one dataset for each of the four flavors in compliance with our live imaging quality control criterion, i.e. the embryos survive imaging and develop into fully functional adults (Strobl et al., 2015), we required 23 attempts (5, 7, 5, and 6 for the four flavors, respectively) in total. Based on these results, and with respect to our previous live imaging-based studies, in which we had hardly any issues when we started recording after blastoderm formation (Strobl and Stelzer, 2014; Strobl et al., 2018), we conclude that *Tribolium* is disproportionally susceptible to light during very early embryogenesis.

### Advantages of ACOS and AGOC/ACOS

Both ACOS and AGOC/ACOS provide the same advantages as AGOC (Strobl et al., 2018). Further, the combined concept is associated with multiple higher-tier benefits of which the most important will be discussed in detail.

Due to the modular design of the concepts, AGOC and ACOS sublines can be freely combined to create exactly those double homozygous hybrid sublines that are best suited to answer the respective scientific questions. So far, sufficient AGOC and ACOS *Tribolium* lines/sublines with various expression patterns and/or intracellular labels for more than twenty differently patterned hybrid sublines are already available, and more will follow in the near future.

As demonstrated, AGOC/ACOS can be conveniently used to manage two transgenes that are located in close proximity on the same autosome and measure their genetic linkage. Evaluation of a total of 4,070 descendants as part of the Gruul #10 mating procedure indicated that the ACOS{ATub′#O(MEM)-mRuby} #2 and AGOC{ATub′H2B-mEmerald} #3 sublines both carry their transgenes on ChLG9 with a genetic linkage of about 8.31 cM. Comparison of our insertion junction sequencing results with a *Tribolium*-associated genome mapping study (Lorenzen et al., 2005) revealed that the ACOS transgene inserted in close proximity (about 0.18 Mbp) upstream of the 32.H10s bacterial artificial chromosome-based genetic marker (GenBank: CZ012621), while the AGOC transgene inserted in very close proximity (about 0.11 Mbp) upstream of the 36.C02t bacterial artificial chromosome-based genetic marker (GenBank: CZ012660.1). The empirically determined linkage between these markers of about 10.2 cM is acceptably close to our result, the deviation possibly derives from the usage of different background strains [GA-2 and ab-2 in the mapping study, PWAS – a derivate of the pearl background strain (Grubbs et al., 2015) – in this study]. Although recombination rates in different strains of *Tribolium* have not yet been systematically compared, such phenomena are known in several *Drosophila* species (Chan et al., 2012; Samuk et al., 2020).

The actual plasmids of our vector concept are primarily designed for creating lines suitable for fluorescence microscopy. Due to the slot-based architecture of the fluorescent protein expression cassette, one or two cloning steps are usually sufficient to obtain an injection-ready plasmid for basically any insect model organism (Figs S1C and S2). This allows a systematic creation of single and double homozygous cultures, which, in turn, produce only descendants that possess the same genotypes as their parents. This ensures that even when progeny is randomly or arbitrarily selected, all chosen individuals will provide fluorescence signal.

### Perspective

Besides application in *Tribolium* and adaptation to other model organisms, we see broad expansion capability for our vector concepts. For example, AGOC/ACOS can be combined with the GAL4/UAS approach, a modular binary expression system usually based on two independent transgenes. The first transgene expresses the GAL4 transcription factor under control of a driver of choice, i.e. a promoter or enhancer, while the second transgene expresses a reporter of choice, e.g. a fluorescent protein, under control of the GAL4-activated UAS. If both transgenes are present within the same organism, the promoter of choice defines the spatio-temporal expression pattern of the reporter (Webster et al., 1988). Several years after the initial study, the system has been adapted for *Drosophila* (Brand and Perrimon, 1993), and today, a vast arsenal of transgenic GAL4 and UAS *Drosophila* lines is available (Caygill and Brand, 2016). Meanwhile, the system has also been adapted for *Tribolium* (Schinko et al., 2010) and vertebrate model organisms such as zebrafish (Halpern et al., 2008). Regarding the experimental workflow, the most simple and thus common approach is to work with double hemizygous individuals obtained from specific parental compositions (Kawakami et al., 2016). However, since respective working cultures are typically composed of driver males and reporter females (Duffy, 2002), or vice versa, they have to be regularly reconstituted from stock cultures to maintain full control over the genotype. In contrast, AGOC/ACOS can now be used for the systematic creation of double homozygous GAL4/UAS transgenic lines that can be kept as continuative cultures for years without any need for recurring reconstitution.

Further, our concepts can be combined with genome engineering techniques such as CRISPR/Cas9 (Gilles and Averof, 2014) for fluorescence microscopy-based analyses of homozygous lethal gene knockout phenotypes, which is currently a cumbersome approach due to the associated extensive transgene management efforts. In such an approach, AGOC is used, as usual, to create homozygous transgenic lines suitable for fluorescence live imaging. In parallel, genome engineering is used to replace an endogenous gene fully or partially with an ACOS-based sequence, and the mating procedure is run until post-recombination hemizygotes are available. Hybridization and two further crosses will result in (mO/mO; mCe) and (mO/mO; mVe) descendants (as well as their respective (mC/mC; mCe) and (mC/mC; mVe) counterparts, which are functionally similar), i.e. individuals that are homozygous for the AGOC-based fluorophore-expressing transgene and hemizygous for the ACOS-based knockout transgene. Consequently, respective descendants are always homozygous for the live imaging-associated transgene, while coincidence of mCe and mVe reveals the share of descendants that are also homozygous for the knockout and, in consequence, show the respective phenotype. Depending on the activity window of the targeted gene, our concept may even allow to identify homozygous knockouts before the phenotype manifests, which in turn would be a convenient approach to image the transition from yet normal to aberrational development. As CRISPR/Cas9-based genome engineering has already been established for *Tribolium* (Gilles et al., 2015), a proof-of-principle study is reasonable in terms of time and effort.

Finally, AGOC/ACOS can be complemented by additional color-shifted concepts to achieve full visual control over three or even more independent transgenes. The expansion potential in this direction is, in theory, only limited by the number of visually clearly distinguishable transformation markers. Even though the demand for multi-transgene management concepts is yet moderate, the point will eventually approach at which no more insights can be obtained from single genetic manipulations. The next higher experimental order are double manipulations, which are much more convenient endeavors when sophisticated transgene management technology is available from the beginning.

## MATERIALS AND METHODS

### *Tribolium castaneum* strains and rearing

The *Tribolium castaneum* (red flour beetle, NCBITaxon:7070) Plain-White-As-Snow (PWAS) background strain was used (Strobl et al., 2018). This strain carries the pearl (Grubbs et al., 2015) and light ocular diaphragm mutations (Mocelin and Stuart, 1996), which result in entirely unpigmented eyes. Cultures were kept in numbers of 200-500 individuals in growth medium [full grain wheat flour (SP061036, Demeter) supplemented with 5% (wt/wt) inactive dry yeast (62-106, Flystuff)] in 1 L glass bottles in a 12 h-light:12 h-darkness cycle at 32°C and 70% relative humidity (DR-36VL, Percival Scientific). All animal-related experiments were approved by the institutional ethics committee (Tierschutzkommission der Goethe-Universität, which is supervised by the Regierungspräsidium Gießen, Hessen, Germany) and performed in agreement with the German Animal Welfare Act (Tierschutzgesetz/Tierschutz-Versuchstierordnung), based on ETS No.123 (European Convention for the Protection of Vertebrate Animals used for Experimental and other Scientific Purposes) and EU Directive 2010/63/EU (European Directive, 2010/63/EU, 2010) as well as the ARRIVE 2.0 guidelines (du Sert et al., 2020). Genes and chromosomes (ChLG) were designated according to the Tcas5.2 assembly (Herndon et al., 2020) of the *Tribolium* genome (Richards et al., 2008).

### General vector design

One of the central elements of this study is the pACOS{#P′#O(MEM)-mRuby} vector (Fig. S1A), whose creation and further usage is described below. The vector contains two fluorescence-based and thus phenotypically distinguishable markers that consist of (i) the artificial 3×P3 promoter (Berghammer et al., 1999), (ii) the open-reading frame for either mCerulean2 (Markwardt et al., 2011) or mVenus (Nagai et al., 2002), and the (iii) SV40 poly(A) (van den Hoff et al., 1993). Both markers are flanked upstream by a LoxP (Hamilton and Abremski, 1984) and downstream by a LoxN (Livet et al., 2007) site, which results in interweaved, but incompatible Lox site pairs (Fig. S1B). Further, it contains a modular fluorescent protein expression cassette, which consists of (i) a two-slot cloning site composed of a promoter (#P) slot and an open-reading frame (#O) slot, (ii) a 9 bp Ala-Ala-Ala linker, (iii) the codon-optimized mRuby2 (Lam et al., 2012) open-reading frame, and (iv) an elongated variant of the SV40 poly(A) (van den Hoff et al., 1993). The #P slot can be accessed by either the AscI / FseI site pair or scarlessly by the head-to-head-oriented double BtgZI site pair. The #O slot carries the open-reading frame for the human *Growth Associated Protein 43* (GAP43) (Denny, 2006) membrane anchor tag (MEM) per default and can be accessed by the FseI / NotI site pair. The mRuby2 open-reading frame can be accessed by the NotI / SbfI site pair (Fig. S1C).

### Molecular biology: genomic and complementary DNA

About 20 PWAS adults (40 mg) were starved for 24 h before genomic DNA was extracted using a dedicated kit (Blood and Tissue Kit, 69504, Qiagen) according to the manufacturer's instructions. Messenger RNA was extracted from about 10 PWAS adults (20 mg) with dedicated reagents (TRIzol, 15596026, Thermo Fisher Scientific) and complementary DNA was transcribed using a suitable reverse transcriptase reaction kit (Superscript III Reverse Transcriptase, 18080093, Thermo Fisher Scientific) in conjunction with random hexamer primers.

### Molecular biology: general information

In this study, 20 vectors (Fig. S2 and Table S9) plus the commercial pGEM-T Easy subcloning vector (A1360, Promega) were used. Seven have been previously described, one was ordered as a gene synthesis plasmid, while all remaining vectors are derivates. A high-fidelity DNA polymerase (Phusion, M0530L, New England BioLabs) was used for all PCRs, and the T4 DNA ligase (M0202L, New England BioLabs or provided with the pGEM-T Easy vector) for all ligations. Cloning primers are listed in Table S10.

### Molecular biology: the promoter and open-reading frame library vectors

Two of the library vectors used in this study, pTC-ATub′-GEM-T Easy and pTC-ATub′H2B-GEM-T Easy, have been previously described (Strobl et al., 2018). The also previously described endogenous *polyubiquitin* promoter (Ub′) sequence (Gene ID: 641546) (Lorenzen et al., 2002) and the *elongation factor 1-alpha* promoter (EFA′) sequence (Gene ID: 655495) (Peel and Averof, 2010; Sarrazin et al., 2012), as well as the *tubulin alpha 1-like protein* open-reading frame (′ATub) sequence (Gene ID: 655491) were amplified from genomic or complementary DNA by using the C1, C2 and C3 extraction PCR primer pairs, respectively. Amplification was followed by A-tailing using a thermostable DNA polymerase (Taq DNA Polymerase, recombinant, 10342020, Thermo Fisher Scientific) and ligation into pGEM-T Easy. The resulting vectors were termed pTC-Ub′-GEM-T Easy, pTC-ShEFA′-GEM-T Easy, and pTC-′ATub-GEM-T Easy.

### Molecular biology: the pACOS{#P′#O(LA)-mEmerald}, pGS[#P′#O(MEM)-mRuby] and pACOS{#P′#O(MEM)-mRuby} vectors

An artificial sequence, consisting of (i) an AvrII site, (ii) mVe and mCe as well as their flanking Lox sites as described above, and (iii) a XhoI site, was excised from pGS[ACOS] (Strobl et al., 2018) with AvrII/XhoI and inserted (in reverse orientation) into the backbone of the accordingly digested pAGOC{#P′#O(LA)-mEmerald} vector (Strobl et al., 2018), replacing mC and mO while the flanking Lox sites remain principally unchanged. The resulting vector was termed pACOS{#P′#O(LA)-mEmerald} and used as an intermediate vector for further cloning operations.

An artificial sequence, consisting of (i) a FseI site, (ii) the modular fluorescent protein expression cassette as described above, and (iii) a SbfI site, was *de novo* synthesized and inserted into the unique SfiI site of pMK-RQ (Thermo Fisher Scientific) by the manufacturer. The resulting vector was termed pGS[#P′#O(MEM)-mRuby]. The sequence was excised with FseI/SbfI and inserted into the backbone of the accordingly digested pACOS{#P′#O(LA)-mEmerald} vector. The resulting vector was termed pACOS{#P′#O(MEM)-mRuby} and used as an intermediate vector for further cloning operations.

### Molecular biology: the pACOS{ATub′#O(MEM)-mRuby} and pACOS{Ub′#O(MEM)-mRuby} vectors

The ATub′ and Ub′ sequences were amplified from the respective library vectors with the C4 and C5 transfer PCR primer pairs, respectively, which introduced upstream an AscI and downstream a BsmBI (ATub′) or BsaI (Ub′) site. The PCR products were digested accordingly, and pACOS{#P′#O(MEM)-mRuby} was digested with BtgZI, which led to compatible overhangs and allowed scarless insertion of the promoter sequences into the #P slot. The resulting vectors were termed pACOS{ATub′#O(MEM)-mRuby} and pACOS{Ub′#O(MEM)-mRuby} and were used for germline transformation.

### Molecular biology: the pACOS{#P′ATub-mRuby} and pACOS{ATub′H2B-mRuby} vectors

The ′ATub sequence was amplified from pTC-′ATub-GEM-T Easy with primer pair C6, which introduced upstream a FseI and downstream a NotI site. The PCR product and pACOS{#P′#O(MEM)-mRuby} were digested accordingly and the product was inserted into the #O slot of the vector. The resulting vector was termed pACOS{#P′ATub-mRuby} and used as an intermediate vector for further cloning operations. The ATub′H2B promoter/open-reading frame sequence was excised from pTC-ATub′H2B-GEM-T Easy with AscI/NotI and inserted into the #P′#O double slot of the accordingly digested pACOS{#P′#O(MEM)-mRuby} vector. The resulting vector was termed pACOS{ATub′H2B-mRuby} and used for germline transformation. Respective transgenic sublines have already been the subject of a previous study (Strobl and Stelzer, 2021).

### Molecular biology: the pACOS{ATub′ATub-mRuby}, pACOS{Ub′ATub-mRuby} and pACOS{ShEFA′ATub-mRuby} vectors

The ATub′ and Ub′ sequences as well as a shortened version of the EFA′ sequence (ShEFA′) were amplified from the respective library vectors with the C7, C8, and C9 transfer PCR primer pairs, respectively, which introduced upstream an AscI and downstream a BsmBI (ATub′ and ShEFA′) or BsaI (Ub′) site. The PCR products and pACOS{#P′ATub-mRuby} were digested accordingly and the product was inserted into the #P slot of the vector. The resulting vectors were termed pACOS{ATub′ATub-mRuby}, pACOS{Ub′ATub-mRuby} and pACOS{ShEFA′ATub-mRuby} and used for germline transformation.

### Molecular biology: the pFIRE{HSP68′NLS-Cre} vector

The pAGOC vector was digested with AflII and AvrII and ran on a 1% (wt/vol) broad range agarose (T846.3, Carl Roth) in TAE buffer gel at 120 V for 20 min. The lower band, which contained the mC sequence, was extracted from the gel and used as a template to amplify mC with the C10 transfer PCR primer pair, which omitted the flanking Lox sites and introduced upstream an AflII and downstream an AvrII site. The PCR product was digested with AflII/AvrII and inserted (in forward orientation) into the backbone of the accordingly digested pICE{ATub′NLS-Cre} vector (Strobl et al., 2018), replacing mCe. The resulting vector was termed pFIRE{ATub′NLS-Cre} and used for germline transformation.

### Germline transformation

Between 700 and 800 F0 PWAS adults were incubated on egg collection medium [405 fine wheat flour (SP061006, Demeter) supplemented with 5% (wt/wt) inactive dry yeast (62–106, Flystuff)] at room temperature (23°C±1°C) in light for 2 h. After the incubation period, the adults were removed, and the eggs (around 500) were extracted from the flour and incubated for another hour, as stated above. The eggs were briefly washed in 10% (vol/vol) sodium hypochlorite (425044–250 ML, Sigma-Aldrich) in autoclaved tap water for 10 s and stored in autoclaved tap water before they were lined up on microscopy slides within the next hour. Injection was performed with a mixture of 500 ng/µl transformation-ready vector and 400 ng/µl pATub′piggyBac (Strobl et al., 2018) in injection buffer (5 mM KCl, 1 mM KH_2_PO_4_ in ddH_2_O, pH 8.8) using a micro-injector (FemtoJet, Eppendorf) and 0.7 µm outer diameter injection capillaries (Femtotips II, Eppendorf) with an injection pressure of 400-800 hPa. After injection, the microscopy slides with embryos were placed on 5 mm high 1% (wt/vol) broad range agarose in tap water ‘platforms’ within Petri dishes and incubated at 32°C. After 3 days, hatched larvae, i.e. F1 potential mosaics, were collected and raised individually in single wells of 24-well plates as described above. Germline transformation resulted [if the previously described ACOS{ATub′H2B-mRuby} #1 to #3 sublines are also taken into account (Strobl and Stelzer, 2021)] in a total of 7 lines with 15 sublines.

### Mating procedure, insert number determination cross and homozygous viability cross

The mating procedures for the systematic creation of homozygous lines and double homozygous hybrid sublines are described within the results section. Generations are numbered using the F-based system to account for the fact that the mating procedure involves (i) sibling crosses and (ii) transgenic hybrid lines. For each crossing step, one female-male pair and typically five backup female-male pairs were mated in small glass vials filled with 1.5 g, 2.5 g (G3 cross), or 3.5 g (G7 and G8 cross) of growth medium. Progeny were scored for marker presence during the larval, pupal, and adult stages by using a fluorescence stereo microscope (SteREO Discovery.V8, Zeiss) with appropriate filter sets as previously described (Strobl and Stelzer, 2021). A one-sample/two-tailed Student's *t*-test was performed to determine whether the arithmetic means differ significantly from the theoretical Mendelian ratios. Insert numbers were estimated by mating F2 hemizygotes with wild types and scoring the progeny, whereby a transgene distribution of 60% or less was interpreted as a single insertion. Homozygous viability was estimated by mating two F3 hemizygous siblings and scoring the progeny, whereby transformation marker presence in more than 70.8% [as the arithmetic mean of the theoretical Mendelian ratios for homozygous viability (75%) and homozygous lethality (66.7%)] of the descendants was interpreted as homozygous viable.

### Determination of insertion junction via inverse PCR

Insertion junctions were determined with the inverse PCR approach (Ochman et al., 1988; Triglia et al., 1988) using dedicated primer pairs (Table S10). At first, inverse PCR was performed for the junction at the 3′ piggyBac terminal repeat with the I-3′ primer pair with several promoter-specific and several 3′-backbone-specific restriction enzymes, and if unsuccessful, also at the 5′ piggyBac terminal repeat with the I-5′ primer pair and several 5′-backbone-specific restriction enzymes. PCR products were purified (NucleoSpin Gel and PCR Clean-up Kit, 740609.250, Macherey-Nagel) and directly sequenced with the I-3′-Seq or I-5′-Seq primers, respectively. For each successful inverse PCR, a control PCR at the respective other side was performed. For the control PCR, location-specific primers were used to perform a standard PCR. The sequencing results were aligned to the recent Tcas5.2 assembly (Herndon et al., 2020) of the *Tribolium* genome (Richards et al., 2008) via the EnsemblMetazoa BLAST (Howe et al., 2020).

### Light sheet fluorescence microscopy

Sample preparation and mounting using the cobweb holder mounting method as well as live imaging using digitally scanned laser light sheet fluorescence microscopy (DSLM) (Keller and Stelzer, 2008, 2010) was performed as previously described (Strobl and Stelzer, 2014; Strobl et al., 2015, 2017a) with the exception that the incubation time in sodium hypochlorite solution was halved from 60 to 30 s. Embryo collection was performed with (i) the F7+ continuative (mCe/mCe) homozygous cultures of the ACOS{ATub′#O(MEM)-mRuby} #1 and #2 sublines, (ii) (mCe) post-recombination hemizygous females of the ACOS{Ub′#O(MEM)-mRuby} #1 and #2 sublines×wild-type male cultures, (iii) the F7+ continuative (mCe/mCe) and (mVe/mVe) homozygous cultures of the ACOS{Ub′ATub-mRuby} #2 subline as well as (iv) the F10+ continuative (mO/mO; mCe/mCe), (mC/mC; mCe/mCe), (mO/mO; mVe/mVe) and (mC/mC; mVe/mVe) double homozygous cultures of the Gruul #8 subline for 1 h at room temperature (23°C±1°C). For single time point imaging, embryos derived from (i) and (ii) were incubated for either 24 h or 48 h at 32°C. For live imaging embryos derived from (iii) and (iv) were incubated for 14 h at 20°C. Sample preparation and mounting took approximately 1 h at room temperature so that embryos derived from (i) and (ii) were either in the middle of germband elongation or in the middle of germband retraction, while embryos derived from (iii) and (iv) were in the middle of blastoderm formation, right before the 10th synchronous division wave. For single time point imaging, embryos were recorded in one channel along four directions. For live imaging, embryos were recorded in one or two channels along one direction with an interval of 6 min. All shown embryos survived the imaging procedure, developed to healthy and fertile adults, and when mated with wild types, produced only transgenic progeny that were also fertile. Comprehensive metadata for the ten live imaging datasets is available with this publication (Table S11).

### Material and data availability

The raw scores for all mating procedure result tables, ordered by transgenic subline/hybrid subline and including the data from our previous study (Strobl and Stelzer, 2021), are provided as an Excel table (.xlsx) in Supplementary File 1. Vectors and transgenic lines are available from our laboratory. Sequence information about the 20 vectors used in this study is provided as GeneBank (.gb) and Geneious files (.geneious) in Supplementary File 2. The ten live imaging datasets and associated machine-readable metadata tables (.xlsx) can be accessed at doi.org/10.5281/zenodo.7564542.

## Supplementary Material

10.1242/biolopen.060015_sup1 Supplementary information
