# Supplementary material for: Next generation marker-based vector concepts for rapid and unambiguous identification of single and double homozygous transgenic organisms
Source: Biol Open. 2023 Oct 19;12(10):bio060015. doi: 10.1242/bio.060015 (PMC10602009; doi:10.1242/bio.060015)
Supplement: Supplementary information [file biolopen-12-060015-s1.pdf]

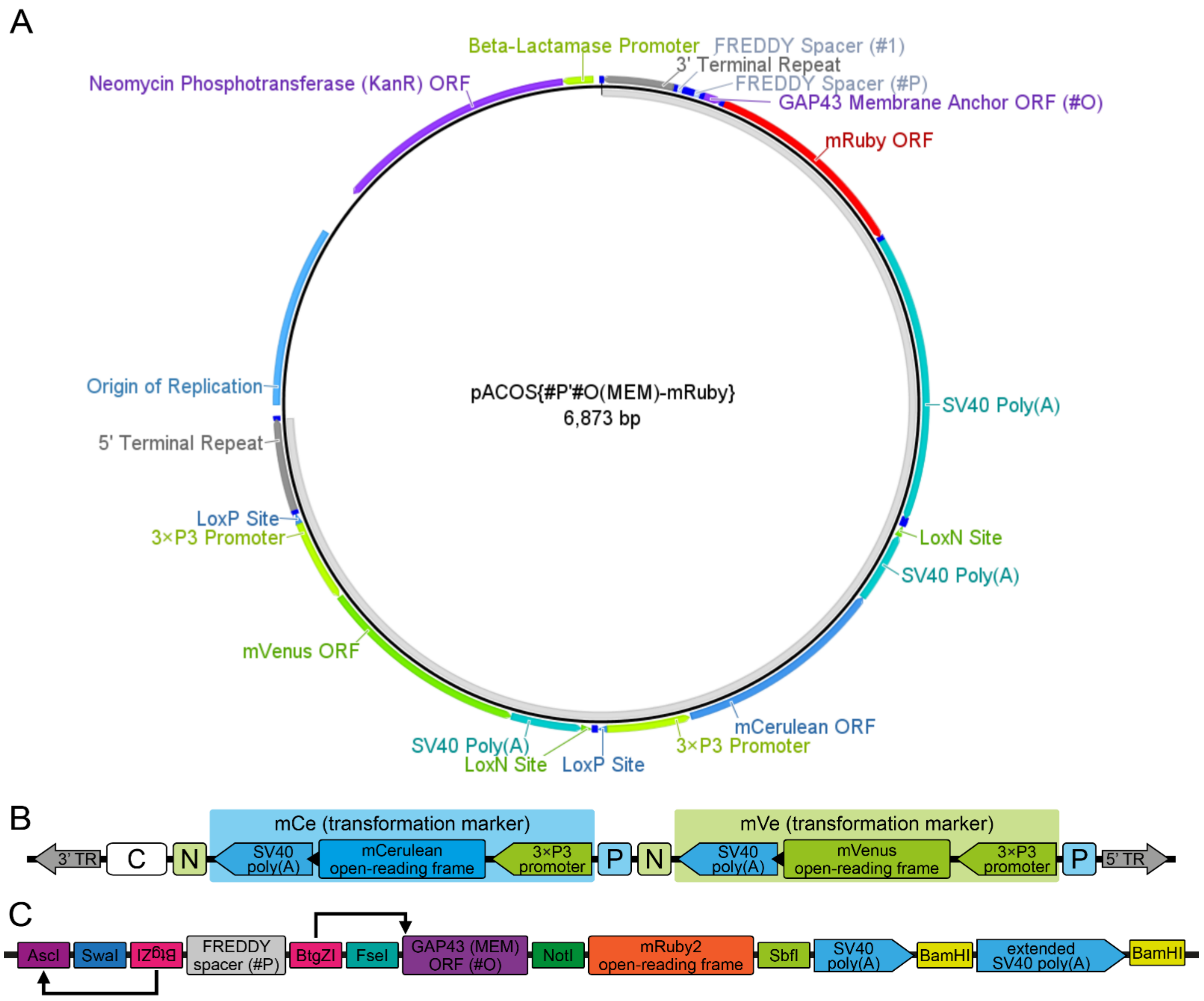

Fig. S1. The pACOS{#P'#O(MEM)-mRuby} vector. (A) Vector map of pACOS{#P'#O(MEM)-mRuby}, which is in principle a color-shifted derivate of pAGOC{#P'#O(LA)-mEmerald (cf. Supplementary File 1). The unlabeled dark blue boxes represent the same restriction enzyme sites as the pAVOIAF{#1–#2–#3–#4} vector (Strobl et al., 2018). The light gray band on the inside indicates the transgene. **(B)** Scheme of mCe and mVe that are embedded into interweaved, but incompatible LoxN and LoxP site pairs. Restriction enzyme sites are not shown. Extents of genetic elements are not to scale. **(C)** Scheme of the #P'#O(MEM)-mRuby two-slot cloning site. To insert a promoter, the #P slot can be accessed by the AscI/FseI site pair, or alternatively by the double BtgZI site pair, which flanks a FREDDY spacer. BtgZI is a type IIS restriction enzyme with a non-palindromic recognition sequence. The enzyme cuts the sequence several bp (10/14) downstream, resulting in a 4 bp sticky end. In this vector, the first BtgZI site (in reverse orientation) opens the AscI site, while the second BtgZI site (in forward orientation) opens the human GAP43 membrane anchor tag (MEM) open-reading frame start codon and the first bp of the subsequent codon (indicated by arrows), which allows scarless insertion of respectively digested promoter sequences. The MEM open-reading frame, which is in #O per default, can be substituted with another open-reading frame to change the intracellular localization by the FseI/NotI site pair, while the mRuby2 open-reading frame can be substituted with another fluorescent protein open-reading frame by the NotI/SbfI site pair. Extents of the genetic elements are not to scale. ORF, open-reading frame.

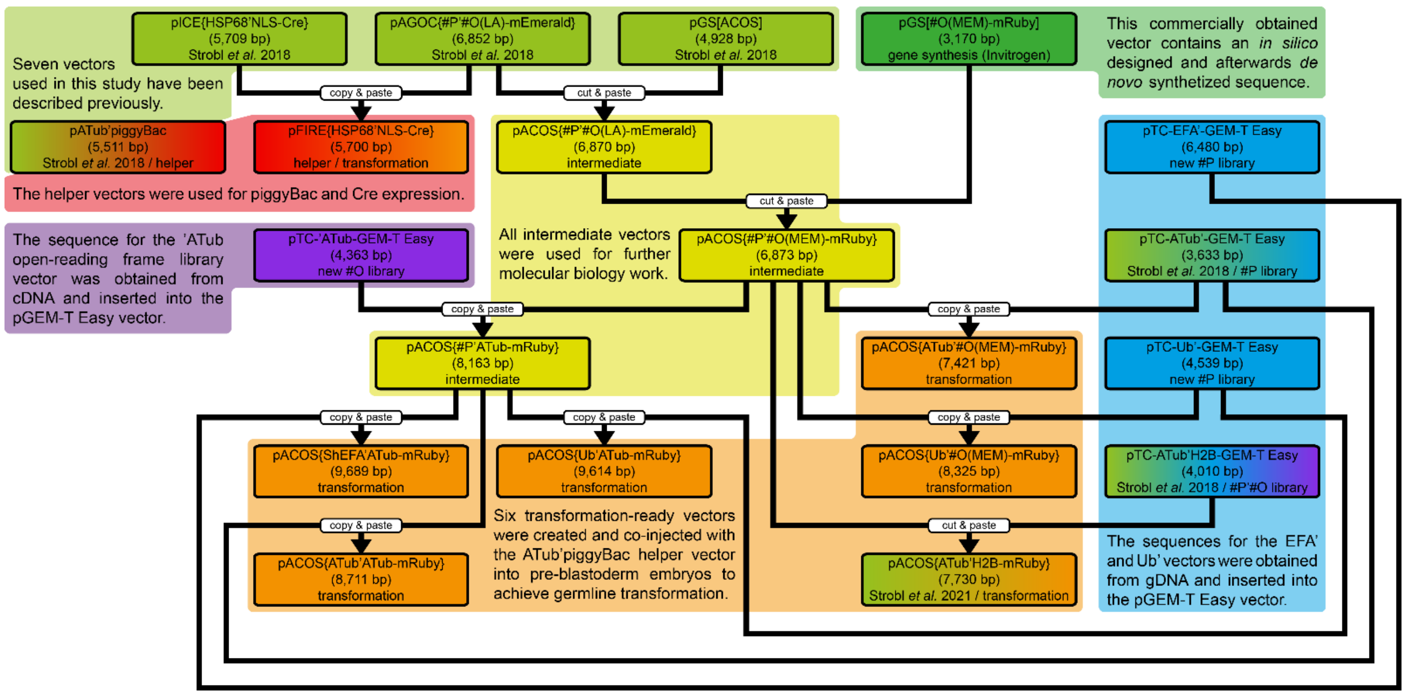

Fig. S2. Development of the twenty vectors used in this study. Each vector belongs to between one and three of seven types, as indicated by the differently colored backgrounds. Light green depicts previously published vectors, dark green depicts gene synthesis vectors, red depicts helper vectors, yellow depicts intermediates, blue depicts promoter (#P) library vectors, purple depicts open-reading frame (#O) library vectors, and orange depicts transformation-ready vectors. The 'copy & paste' boxes indicate a molecular biology-based procedure with PCR-based amplification of the insert, while the 'cut & paste' boxes indicate that the respective insert was extracted from another vector and inserted into the respective backbone without any amplification.

Biology Open • Supplementary information

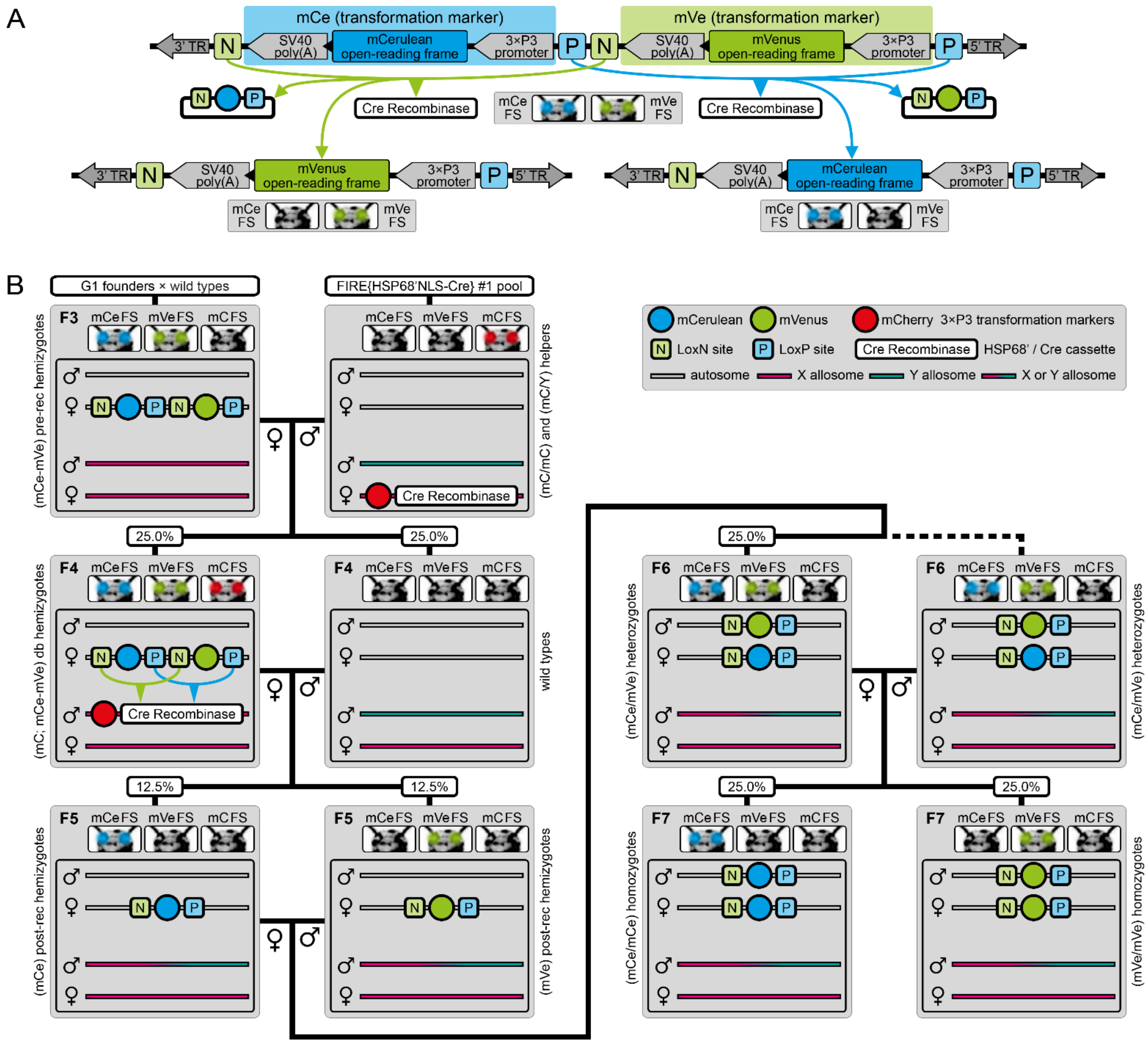

Fig. S3. The ACOS vector concept and associated mating procedure. (A) In ACOS, two fluorescence-based transformation markers, mCe and mVe, are embedded in a piggyBac-based vector characterized by 3' and 5' terminal repeats (TR) necessary for genomic insertion. Both markers consist of the eye-specific 3×P3 promoter, the open-reading frame for the respective fluorescent protein, *i.e.* mCerulean or mVenus, as well as the Simian Virus 40 (SV40) poly(A) and can thus be detected and distinguished by using appropriate spectral filter sets. Further, both are flanked upstream by a LoxP and downstream by a LoxN site. Consequently, Cre-mediated recombination excises only one marker from the transgene as the remaining single LoxP and LoxN sites are incompatible. Individuals expressing the Cre recombinase in germline cells give rise to progeny that carry only one marker. **(B)** The ACOS-associated F3 to F7 mating procedure for the systematic creation of homozygous transgenic lines. A rounded rectangle illustrates the genotype, gray bars represent the ACOS transgene location on an autosome, pink bars represent the Cre recombinase-expressing helper transgene location on the X allosome, and turquoise bars represent the Y allosome. The percentage boxes indicate the theoretical ratios of the progeny that carry the respective genotype, the dashed line represents genotypically identical siblings. FS, filter set; rec, recombination; db, double.

Biology Open • Supplementary information

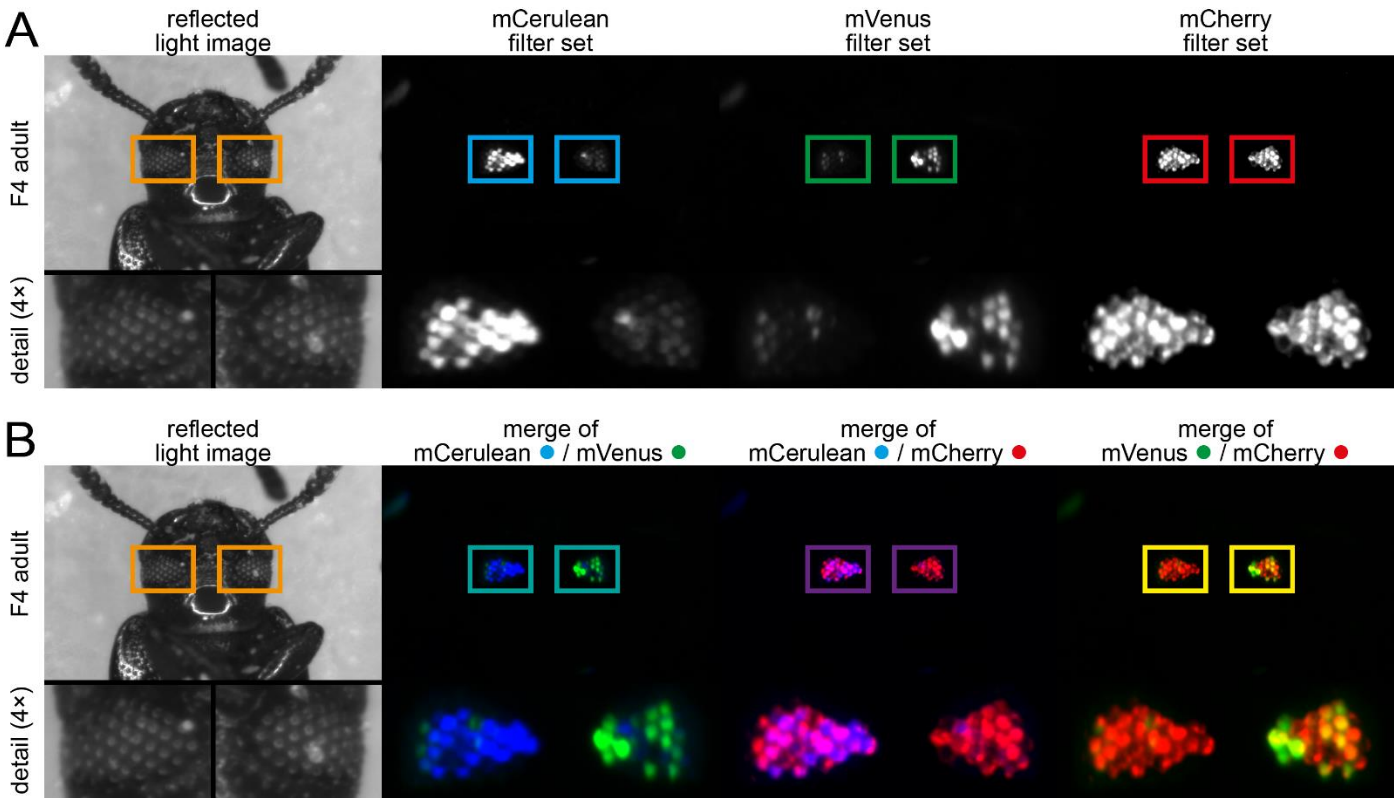

Fig. S4. The F4 (mC; mCe-mVe) double hemizygous generation. (A) The F4 hybrids are hemizygous for both the ACOS transgene, which carries both mCe and mVe embedded into interweaved, but incompatible Lox site pairs, and the helper transgene, which carries mC and the Cre recombinase expression cassette. Within this cassette, expression of a nuclear-localized Cre recombinase is mediated by the endogenous *heat shock protein 68b* promoter. During the development of the F4 generation from the zygote to the fertile adult, this promoter exhibits a slight leaky expression. Over time, this leads to recombination in germ cells (*i.e.* the prerequisite for the ACOS vector concept procedure) but also in somatic progenitor cells that later on give rise to distinct, typically spatially clustered cell populations. This effect is evident in the compound eyes of adult beetles in all ACOS sublines. **(B)** Within the merged images, it becomes evident that certain clusters of ommatidia express either only mCe or only mVe.

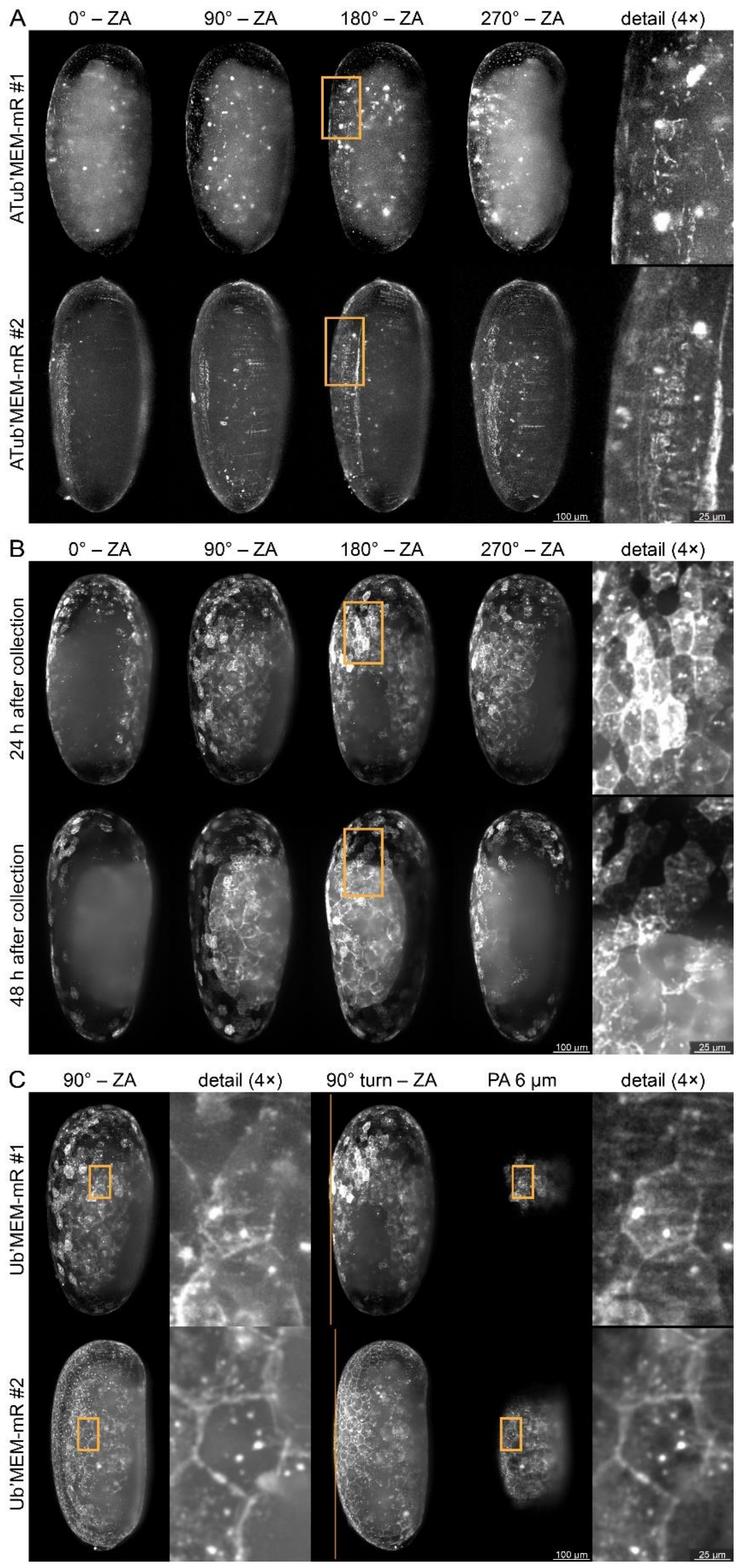

Fig. S5. Single time point imaging of ACOS{ATub'#O(MEM)-mRuby} and ACOS{Ub'#O(MEM)-mRuby} embryos using light sheet fluorescence microcopy. These sublines express the mRuby2-labeled GAP43 membrane anchor tag (MEM) under control of either the *tubulin alpha 1-like protein* or the *polyubiquitin* promoter. **(A)** Comparison of (mCe/mCe) homozygous embryos from the ACOS{ATub'#O(MEM)-mRuby} #1 and #2 sublines after 24 h of incubation at 32°C along four directions. In both sublines, fluorescence patterns characteristic for membrane labeling cannot be recognized. **(B)** Comparison of (mCe) hemizygous embryos from the ACOS{Ub'#O(MEM)-mRuby} #1 subline after 24 h and 48 h of incubation at 32°C along four directions. In this subline, fluorescence patterns characteristic of membrane labeling are evident, but remarkably patchy and almost exclusively found in the serosa (24 h and 48 h) as well as in the yolk sac (48 h). **(C)** Comparison of (mCe/-) hemizygous embryos from the ACOS{Ub'#O(MEM)-mRuby} #1 and #2 sublines after 24 h of incubation at 32°C along four directions. Similar to the #1 subline, fluorescence patterns characteristic of membrane labeling are evident in the #2 subline. The patterns are less patchy than in the #1 subline, but also almost exclusively found in the serosa. The locations of the planes are indicated by the orange lines. ZA, Z maximum projection with intensity adjustment; PA, single plane with intensity adjustment.

Biology Open • Supplementary information

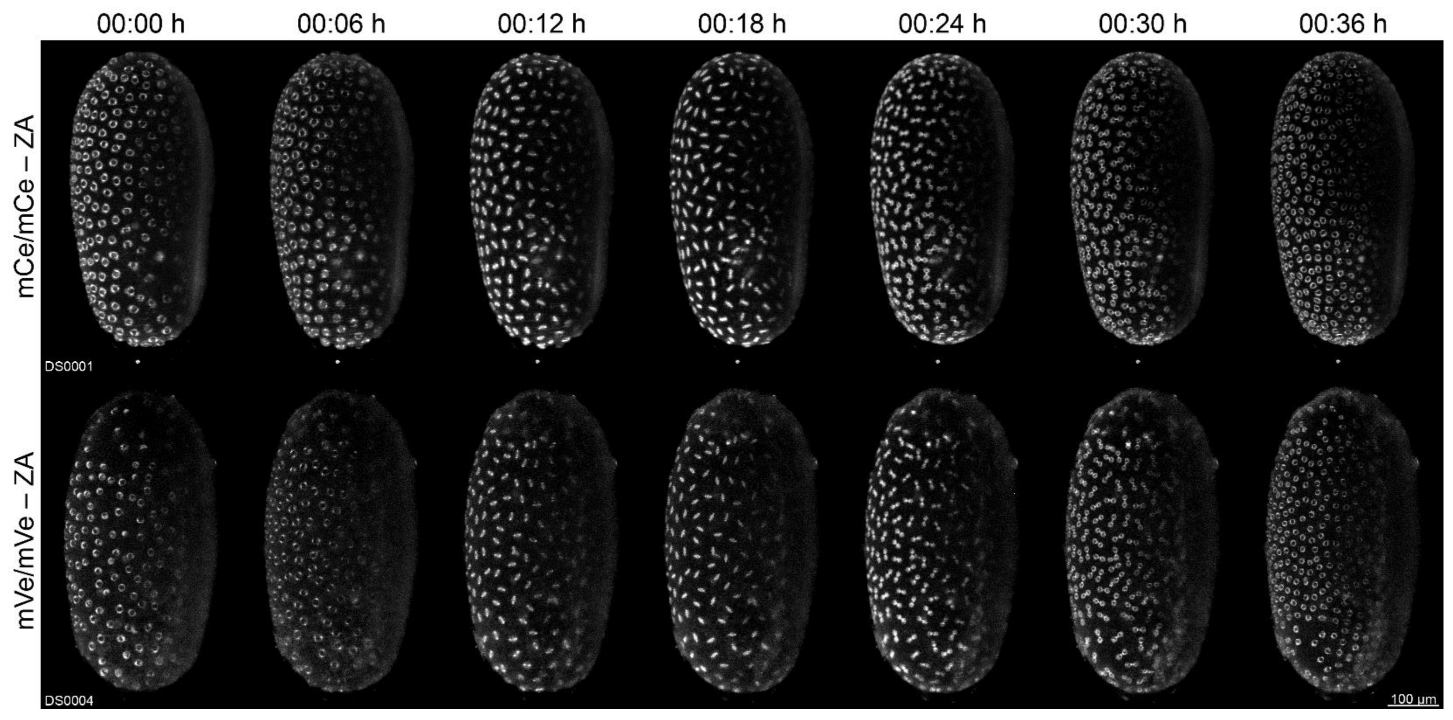

Fig. S6. Fluorescence live imaging of homozygous ACOS{Ub'ATub-mRuby} #2 embryos during blastoderm formation using light sheet fluorescence microcopy. This subline expresses mRuby2-labeled *tubulin alpha 1-like protein* under control of the *polyubiquitin* promoter. Comparative time series of a (mCe/mCe) and (mVe/mVe) embryo proceeding through the 10th synchronous division wave. Both flavors show similar fluorescence patterns. ZA, Z maximum projection with intensity adjustment.

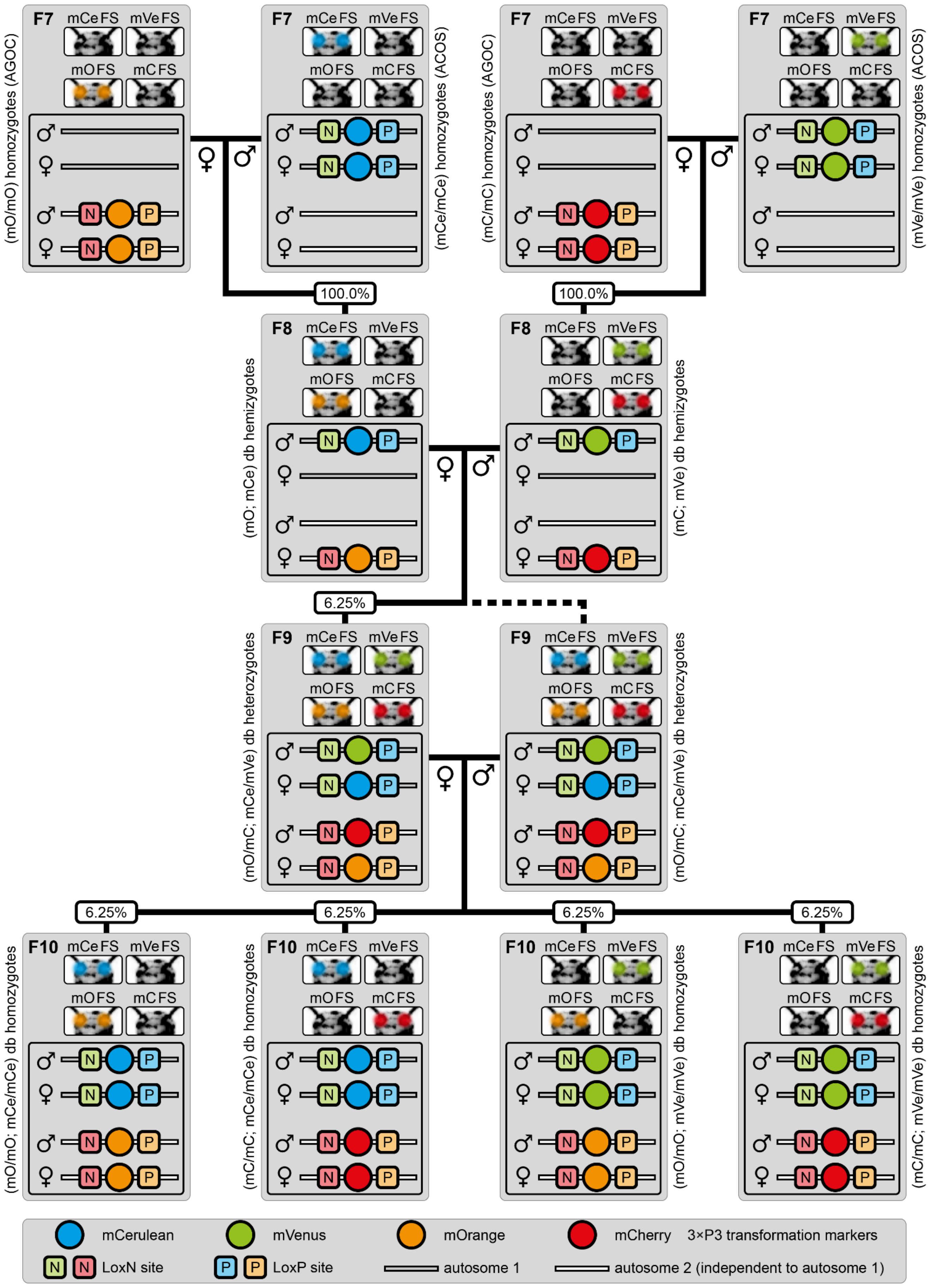

Fig. S7. The AGOC/ACOS-associated F7 to F10 mating procedure for a systematic creation of double homozygous transgenic hybrid sublines. A rounded rectangle illustrates the genotype where white bars represent the AGOC transgene location on one autosome and gray bars represent the ACOS transgene location on a different, *i.e.* independent, autosome. The percentage boxes indicate the theoretical Mendelian ratios of the progeny that carry the respective genotype, the dashed line represents genotypically identical siblings. FS, filter set; rec, recombination; db, double.

Biology Open • Supplementary information

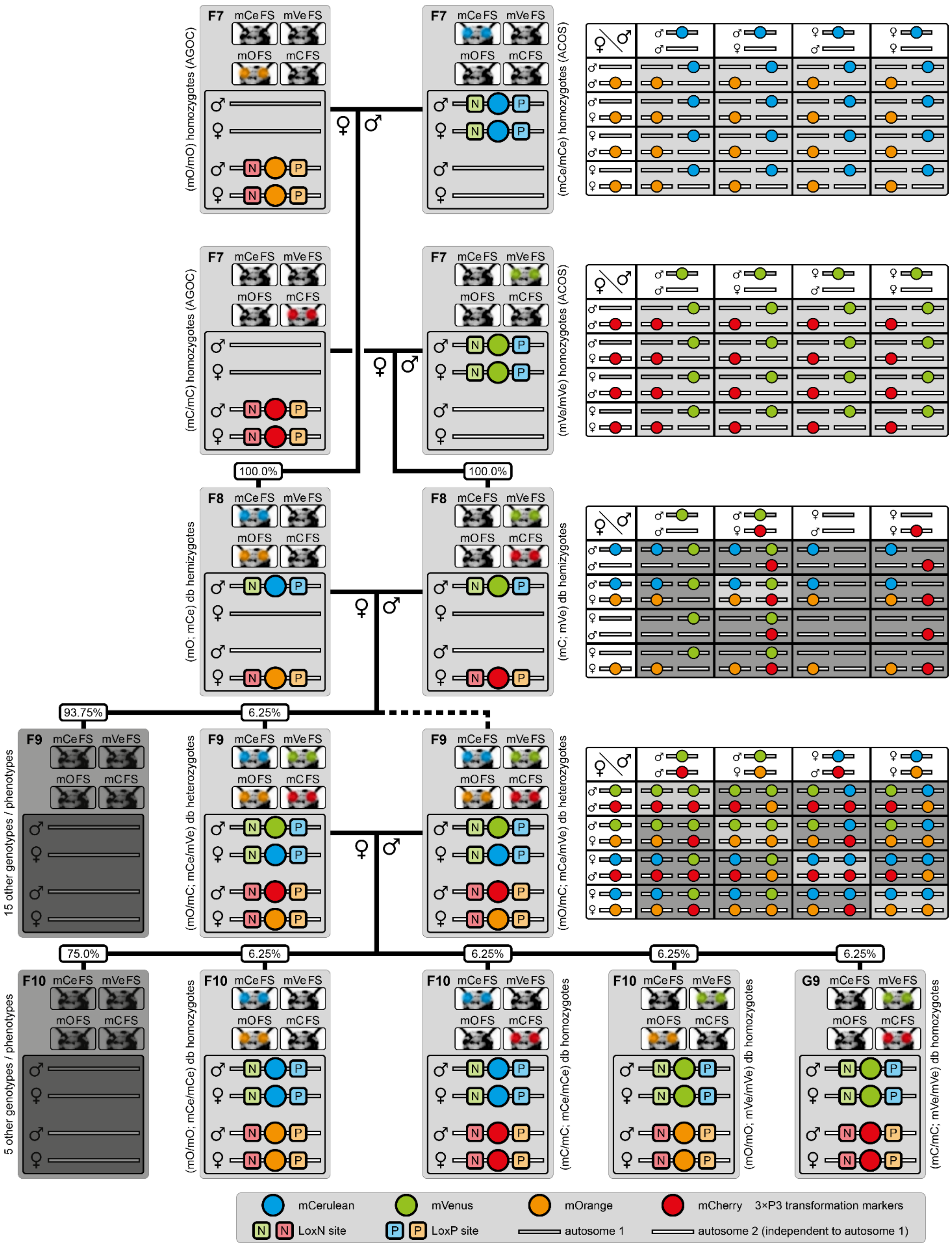

Fig. S8. The AGOC/ACOS-associated F7 to F10 mating procedure with additional outcomes and respective Punnett squares. A light gray background indicates either F7 to F9 outcomes that are used further within the procedure or the final four flavors of F10 homozygotes, while a dark gray background indicates progeny that can be disregarded. A rounded rectangle illustrates the genotype for two independent autosomes, where white bars represent the AGOC transgene location and gray bars represent ACOS transgene location on a different, and thus independent, autosome. The percentage boxes indicate the theoretical ratios of the progeny that carry the respective genotype, the dashed line represents genotypically identical siblings. For convenience, the Punnett squares for the F7 to F9 crosses are provided on the right. FS, filter set; rec, recombination; db, double.

Biology Open • Supplementary information

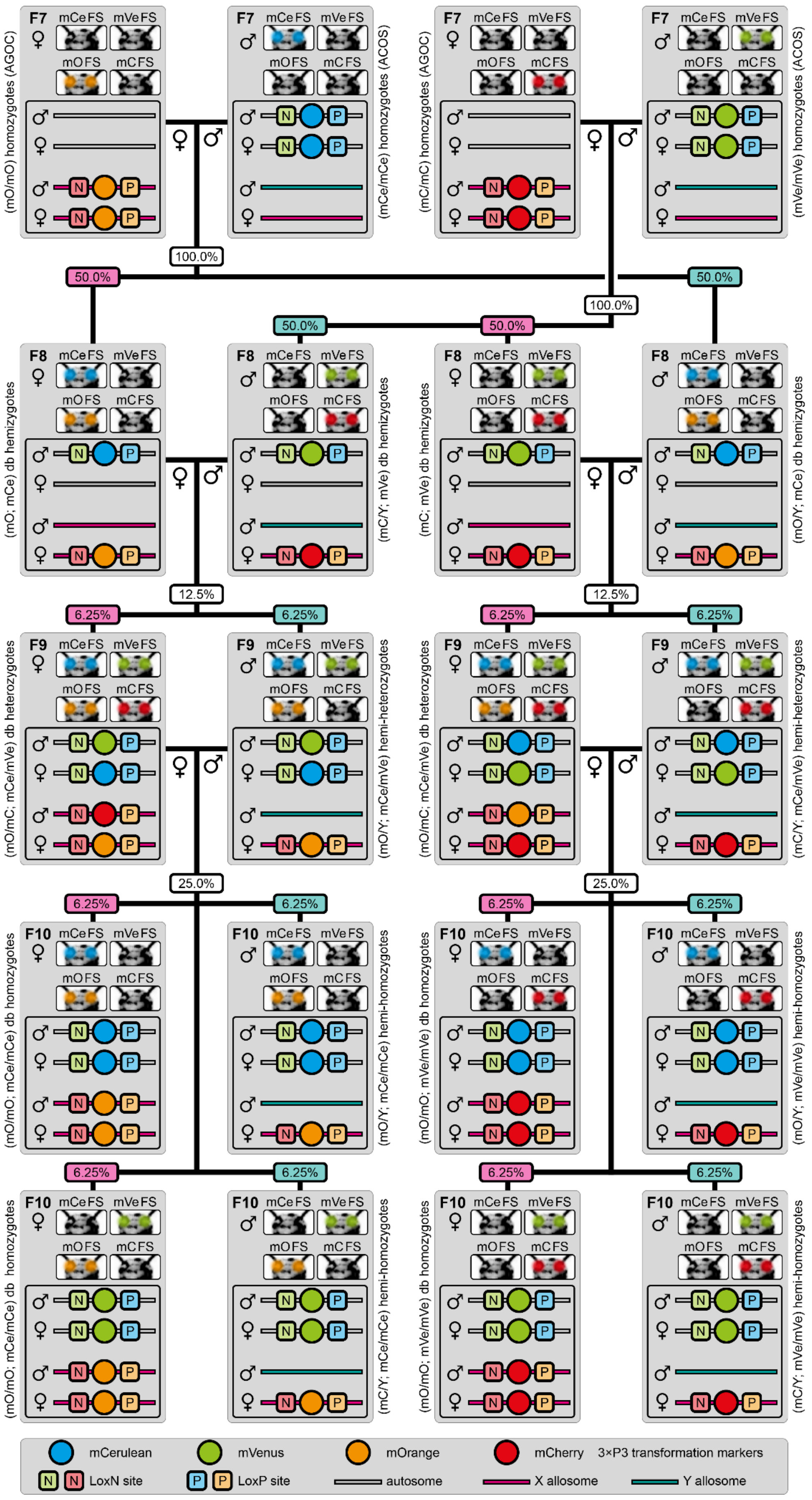

Fig. S9. The AGOC/ACOS-associated F7 to F10 mating scheme for the systematic creation of double homozygous transgenic lines if one transgene is located on the X allosome. A rounded rectangle illustrates the genotype where pink bars represent the AGOC transgene location on the X allosome. Turquoise bars represent the Y allosome and gray bars represent the ACOS transgene location on an autosome. The percentage boxes indicate the theoretical ratios of the progeny that carry the respective genotype. Pink boxes represent female progeny, turquoise boxes represent male progeny. FS, filter set; rec, recombination; db, double.

Biology Open • Supplementary information

Table S1. Insert number and homozygous viability estimation assays for all seventeen ACOS sublines.

Transgenic line	Subline	Insert number estimation assay			Homozygous viability estimation assay		
		●●	●●	total	●●	●●	total
**ACOS{ATub'#O(MEM)-mRuby}**	**#1**	46.1% (71)	53.9% (83)	154	23.9% (28)	**76.1% (89)**	117
	**#2**	44.9% (40)	55.1% (49)	89	15.6% (10)	**84.4% (54)**	64
**ACOS{Ub'#O(MEM)-mRuby}**	**#1**	58.8% (80)	41.2% (56)	136	29.6% (38)	70.4% (38)	54
	**#2**	55.4% (62)	44.6% (50)	112	30.6% (87)	69.4% (197)	284
	**#3**	47.4% (66)	42.6% (49)	115	70.0% (49)	30.0% (21)	70
	**#4**	56.2% (45)	43.8% (35)	80	30.2% (16)	69.8% (37)	53
	**#5**	62.1% (54)	37.9% (33)	87	44.2% (23)	55.8% (29)	52
	**#6**	48.5% (32)	51.5% (34)	66	36.2% (34)	63.8% (60)	94
**ACOS{ATub'H2B-mRuby}**	**#1**	42.9% (21)	57.1% (28)	49	22.7% (10)	**77.3% (34)**	44
	**#2**	46.7% (91)	53.3% (104)	195	29.0% (31)	**71.0% (76)**	107
	**#3**	50.9% (57)	49.1% (55)	112	24.8% (26)	**75.2% (79)**	105
**ACOS{ATub'ATub-mRuby}**	#1	50.8% (66)	49.2% (64)	130	29.9% (41)	70.1% (96)	137
	**#2**	41.9% (26)	58.1% (36)	62	24.8% (28)	**75.2% (85)**	113
**ACOS{Ub'ATub-mRuby}**	**#1**	46.0% (70)	54.0% (82)	152	26.4% (38)	**73.6% (106)**	144
	**#2**	47.4% (45)	52.6% (50)	95	21.6% (25)	**78.4% (91)**	116
**ACOS{ShEFA'ATub-mRuby}**	**#1**	46.4% (32)	53.6% (37)	69	34.2% (14)	65.7% (27)	41
	**#2**	44.7% (51)	55.3% (63)	114	25.8% (17)	**74.2% (49)**	66

Sublines for which the homozygous viability estimation assay resulted in more than 70.8% (marked bold) were assumed to be homozygous viable. The numbers in brackets and in the 'total' sub-columns indicate the number of scored individuals. The ACOS{ATub'H2B-mRuby} sublines have been previously described (Strobl and Stelzer 2021, *Scientific Reports* (doi.org/10.1038/s41598-021-94288-0))

Table S2. Insertion junctions. In the Junction column, the piggyBac TTAA insertion/excision target sequence is marked bold, while differences to the Tcas5.2 assembly (Herndon et al., 2020) are underlined.

Subline	ChLG	Junction	Neighbors	Inverse primer pair and restriction enzyme, control primer pair
ACOS{ATub'#O(MEM)-mRuby}#1	3	5'-AGAGAGGAAACTTAAAATTAATTCTTAT**TTAA**AAAAATATAAGCCATATCATGCAAGCAC-3'	Close to *Ubiquitin carboxyl-terminal hydrolase 10-like Protein* (TC032475) / Approximately 0.5 kbp from Exon 1	I-5' with AgeI, I1
ACOS{ATub'#O(MEM)-mRuby}#2	9	5'-GCTGGAGATACGATCAGAGTCTAAGCTC**TTAA**ATAAACAAGAGACAAGACTCGGAATTCT-3'	Intron 2 of Uncharacterized Gene (TC034345) / Approximately 4.5 kbp from Exon 2 and 15.3 kbp from Exon 3	I-3' with SpeI, I2
ACOS{Ub'#O(MEM)-mRuby}#1	Not X or Y	N.A.	N.A.	N.A.
ACOS{Ub'#O(MEM)-mRuby}#2	Not X or Y	N.A.	N.A.	N.A.
ACOS{Ub'#O(MEM)-mRuby}#3	Not X or Y	N.A.	N.A.	N.A.
ACOS{Ub'#O(MEM)-mRuby}#4	Not X or Y	N.A.	N.A.	N.A.
ACOS{Ub'#O(MEM)-mRuby}#5	Not X or Y	N.A.	N.A.	N.A.
ACOS{Ub'#O(MEM)-mRuby}#6	Not X or Y	N.A.	N.A.	N.A.
ACOS{ATub'H2B-mRuby}#1	6	5'-CAGCAACGATATCAAAAAGCGCGTTAAC**TTAA**TAAGCCATCGCGTCCAATATGTCTTCTT-3'	Intron 2 of *Relaxin receptor 2-like Protein* (TC033440) / Approximately 2.5 kbp from Exon 2 and 8.4 kbp from Exon 3	I-5' with BsrGI, I3
ACOS{ATub'H2B-mRuby}#2	7	5'-GGCATGTGGAACAAATAAGCAAAAGGAT**TTAA**TGATTATTCAAAAATGCTTAATATACTA-3'	Intron 1 of *Protein dead ringer-like Protein* (TC033868) / Approximately 33.0 kbp from Exon 1 and 0.5 kbp from Exon 2	I-3' with PstI, I4
ACOS{ATub'H2B-mRuby}#3	Not X or Y	N.A.	N.A.	N.A.
ACOS{ATub'ATub-mRuby}#1	2	5'-TAATCTTGCGCCAGGGACTCCTTGTTGG**TTAA**TGAATTGTTTTGGACTTTAATACTTTCT-3'	Close to Uncharacterized Gene (TC000226) / Approximately 0.2 kbp from Exon 1	I-3' with SpeI, I5
ACOS{ATub'ATub-mRuby}#2	6	5'-TTGCCCCAATATATTGAACGCAGGTTTA**TTAA**TTTAGTTGAAATGAAGTTACAACAATTT-3'	Intron 1 of *Putative multidrug resistance-associated protein lethal(2)03659-like Protein* (TC014880) / Approximately 2.2 kbp from Exon 1 and 0.5 kbp from Exon 2	I-3' with HindIII, I6
ACOS{Ub'ATub-mRuby}#1	9	5'-CAGTACCTACAAAATTTTCATGTATTTT**TTAA**ATGCGGTTTGTTAGAGAAATAAATAAAA-3'	Intron 1 of *4-hydroxybutyrate coenzyme A transferase-like Protein* (TC034329) / Approximately 2.1 kbp from Exon 1 and 2.2 kbp from Exon 2	I-3' with BssHI, I7
ACOS{Ub'ATub-mRuby}#2	8	5'-TTACCCAATTTGTTTTTATTTTACTGTT**TTAA**TAACTAATTTATTTTTTAGTGTCTATGT-3'	Intron 2 of Uncharacterized Gene (TC006953) / Approximately 0.2 kbp from Exon 2 and 2.6 kbp from Exon 3	I-3' with PciI, I8
ACOS{ShEFA'ATub-mRuby}#1	Not X or Y	N.A.	N.A.	N.A.
ACOS{ShEFA'ATub-mRuby}#2	3	5'-GACCCAGAGAGGTTATGTCAATATGTTT**TTAA**AGTACCCTGAATGTTTATTTTTATGT-3'	Exon1 of *TBC1 domain family member 13-like Protein* (TC003977)	I-5' with EcoRI, I9

Biology Open • Supplementary information

Table S3. F6- and F7-associated control crosses results for the ten homozygous viable homozygous ACOS sublines.

Cross	Genotypes	Line	Subline	Progeny			
				●●●●	●●●●	other	total
F6-S	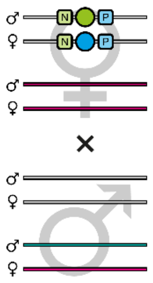	Theoretical	-	50.0%	50.0%	-	-
		ACOS{ATub’#O(MEM)-mRuby}	#1	50.5% (48)	49.5% (47)	-	95
			#2	53.8% (42)	46.2% (36)	-	78
		ACOS{ATub’H2B-mRuby}	#1	51.7% (61)	48.3% (57)	-	118
			#2	53.7% (29)	46.3% (25)	-	54
			#3	56.0% (51)	44.0% (40)	-	91
		ACOS{ATub’ATub-mRuby}	#1	41.0% (41)	59.0% (59)	-	100
			#2	57.3% (67)	42.7% (50)	-	117
		ACOS{Ub’ATub-mRuby}	#2	55.3% (47)	44.7% (38)	-	85
		ACOS{ShEFA’ATub-mRuby}	#1	38.6% (27)	61.4% (43)	-	70
			#2	38.5% (30)	61.5% (48)	-	78
		Mean ± SD	-	49.6% ± 4.7%	50.4% ± 7.4%	-	88.6
		Significance	-	n.s.	n.s.	-	-
F7-mCe	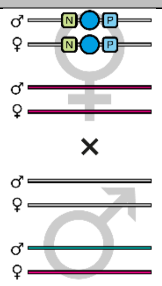	Theoretical	-	100%	-	-	-
		ACOS{ATub’#O(MEM)-mRuby}	#1	100% (88)	-	-	88
			#2	100% (50)	-	-	50
		ACOS{ATub’H2B-mRuby}	#1	100% (45)	-	-	45
			#2	100% (51)	-	-	51
			#3	100% (61)	-	-	61
		ACOS{ATub’ATub-mRuby}	#1	100% (75)	-	-	75
			#2	100% (50)	-	-	50
		ACOS{Ub’ATub-mRuby}	#2	100% (96)	-	-	96
		ACOS{ShEFA’ATub-mRuby}	#1	100% (104)	-	-	104
			#2	100% (71)	-	-	71
		Mean	-	100%	-	-	69.1
		Significance	-	n.s.	-	-	-
F7-mVe	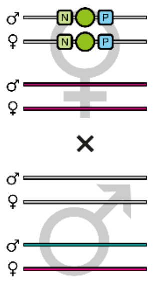	Theoretical	-	-	100%	-	-
		ACOS{ATub’#O(MEM)-mRuby}	#1	-	100% (91)	-	91
			#2	-	100% (48)	-	48
		ACOS{ATub’H2B-mRuby}	#1	-	100% (87)	-	87
			#2	-	100% (80)	-	51
			#3	-	100% (83)	-	83
		ACOS{ATub’ATub-mRuby}	#1	-	100% (70)	-	70
			#2	-	100% (52)	-	52
		ACOS{Ub’ATub-mRuby}	#2	-	100% (68)	-	68
		ACOS{ShEFA’ATub-mRuby}	#1	-	100% (87)	-	87
			#2	-	100% (97)	-	97
		Mean	-	-	100%	-	73.4
		Significance	-	-	n.s.	-	-

The numbers in brackets and in the ‘total’ sub-column indicate the number of scored individuals. No significant differences between the arithmetic means and the theoretical ratios were found. SD, standard deviation; n.s., not significant (*P*>0.05).

Biology Open • Supplementary information

Table S4. Mating procedure results for the seven homozygous lethal ACOS sublines from the F3 to the F6 generation.

Cross	Genotypes	Line	Subline	Progeny								
												total
**F3**	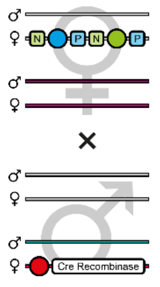	**ACOS{Ub'#O(MEM)-mRuby}**	**#1**	41.8% (46)	-	-	43.7% (48)	1.8% (2)	-	-	**12.7% (14)**	110
			**#2**	33.3% (10)	-	-	20.0% (6)	30.0% (9)	-	-	**16.7% (5)**	30
			**#3**	Not assayed due to the result of the homozygous viability estimation assay (cf. Table 1).								
			**#4**	52.7% (29)	-	-	40.0% (22)	1.8% (1)	-	-	**2.2% (3)**	55
			**#5**	60.0% (12)	-	-	5.0% (1)	15.0% (3)	-	-	**20.0% (4)**	20
			**#6**	30.6% (22)	-	-	25.0% (18)	30.6% (22)	-	-	**13.8% (10)**	72
		**ACOS{Ub'ATub-mRuby}**	**#1**	28.4% (23)	-	-	28.4% (23)	19.7% (16)	-	-	**23.5% (19)**	81
**F4**	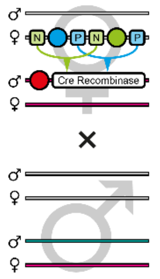	**ACOS{Ub'#O(MEM)-mRuby}**	**#1**	24.5% (25)	**13.7% (14)**	**10.8% (11)**	30.4% (31)	-	7.8% (8)	12.8% (13)	-	102
			**#2**	25.0% (21)	**21.4%% (18)**	**9.5% (8)**	35.7% (30)	-	7.2% (6)	1.2% (1)	-	84
			**#3**	See entry in the respective F3 row.								
			**#4**	51.9% (27)	**0.0% (0)**	**0.0% (0)**	46.2% (24)	-	1.9% (1)	0.0% (0)	-	52
			**#5**	36.8% (21)	**3.5% (2)**	**0.0% (0)**	29.8% (17)	1.8% (1)	26.3% (15)	1.8% (1)	-	57
			**#6**	32.9% (49)	**15.4% (23)**	**5.4% (8)**	25.5% (38)	1.4% (2)	15.4% (23)	4.0% (6)	-	149
		**ACOS{Ub'ATub)-mRuby}**	**#1**	21.5% (14)	**10.8% (7)**	**9.2% (6)**	29.2% (19)	-	15.4% (10)	13.9% (9)	-	65
**F5**	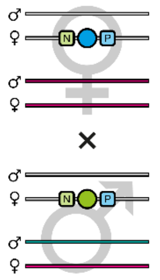	**ACOS{Ub'#O(MEM)-mRuby}**	**#1**	38.2% (26)	22.1% (15)	39.7% (27)	-	**0.0% (0)**	-	-	-	68
			**#2**	29.0% (18)	29.0% (18)	42.0% (26)	-	**0.0% (0)**	-	-	-	62
			**#3**	See entry in the respective F3 row.								
			**#4**	Not assayed since the F4 cross resulted in inconvenient progeny.								
			**#5**	Not assayed since the F4 cross resulted in inconvenient progeny.								
			**#6**	46.7% (14)	16.7% (5)	23.3% (7)	-	**13.3% (4*)**	-	-	-	30
		**ACOS{Ub'ATub-mRuby}**	**#1**	31.4% (27)	31.4% (27)	37.2% (32)	-	**0.0% (0)**	-	-	-	86
**F6**	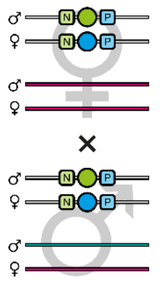	**ACOS{Ub'#O(MEM)-mRuby}**	**#1**	Not assayed since the F5 cross resulted in inconvenient progeny.								
			**#2**	Not assayed since the F5 cross resulted in inconvenient progeny.								
			**#3**	See entry in the respective F3 row.								
			**#4**	See entry in the respective F4 row.								
			**#5**	See entry in the respective F4 row.								
			**#6**	The four F6 (mCe/mVe) heterozygous individuals deceased before pupation.								
		**ACOS{Ub'ATub-mRuby}**	**#1**	Not assayed since the F5 cross resulted in inconvenient progeny.								

Progeny used in the subsequent crosses are marked bold. The numbers in brackets and in the 'total' sub-column show the number of scored individuals. Due to the non-conformal results, we refrained from indicating arithmetic means and standard deviations. * All four (mCe/mVe) heterozygotes died before pupation.

Biology Open • Supplementary information

Table S5. AGOC/ACOS subline pairs.

AGOC subline		ACOS subline		Hybrid subline
**Subline name**	**ChLG**	**Subline name**	**ChLG**	
AGOC{Zen1'#O(LA)-mEmerald} #1	3	ACOS{ATub'H2B-mRuby} #1	6	Gruul #1*
AGOC{Zen1'#O(LA)-mEmerald} #1	3	ACOS{ATub'H2B-mRuby} #2	7	Gruul #2*
AGOC{Zen1'#O(LA)-mEmerald} #1	3	ACOS{ATub'H2B-mRuby} #3	Not X or Y	Gruul #3*
AGOC{ATub'H2B-mEmerald} #3	9	ACOS{ATub'#O(MEM)-mRuby} #1	3	Gruul #4
AGOC{ATub'#O(LA)-mEmerald} #1	2	ACOS{ATub'H2B-mRuby} #1	6	Gruul #5
AGOC{ATub'#O(LA)-mEmerald} #1	2	ACOS{ATub'H2B-mRuby} #2	7	Gruul #6
AGOC{ARP5'#O(LA)-mEmerald} #1	9	ACOS{ATub'H2B-mRuby} #1	6	Gruul #7
AGOC{ATub'H2B-mEmerald} #3	9	ACOS{ShEFA'ATub-mRuby} #2	3	Gruul #8
AGOC{ATub'H2B-mEmerald} #3	9	ACOS{ATub'#O(MEM)-mRuby} #2	9	Gruul #9
AGOC{Zen1'#O(LA)-mEmerald} #1	X	ACOS{ATub'H2B-mRuby} #2	7	Gruul #10

* The Gruul #1 to #3 sublines have been previously described (Strobl and Stelzer 2021, *Scientific Reports* (doi.org/10.1038/s41598-021-94288-0))

Biology Open • Supplementary information

Table S6. F9- and F10-associated control cross results for the five Gruul #4 to #8 sublines.

Cross	Genotypes	Subline	Progeny					
							other	total
**F9-S**	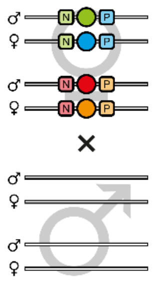	**Theoretical**	25.0%	25.0%	25.0%	25.0%	-	-
		**Gruul #4**	19.7% (40)	19.3% (39)	30.5% (62)	30.5% (62)	-	203
		**Gruul #5**	28.4% (53)	25.1% (47)	20.3% (38)	26.2% (49)	-	187
		**Gruul #6**	25.3% (23)	30.7% (28)	24.2% (22)	19.8% (18)	-	91
		**Gruul #7**	22.0% (24)	22.9% (25)	24.8% (27)	30.3% (33)	-	109
		**Gruul #8**	27.8% (42)	20.5% (31)	29.8% (45)	21.9% (33)	-	151
		**Mean ± SD**	24.7% ± 3.7%	23.7% ± 4.5%	23.9% ± 3.9%	25.7% ± 4.8%	-	148.2
		**Significance**	n.s.	n.s.	n.s.	n.s.	-	-
**F10-mO-mCe**	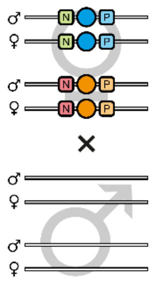	**Theoretical**	100%	-	-	-	-	-
		**Gruul #4**	100% (122)	-	-	-	-	122
		**Gruul #5**	100% (104)	-	-	-	-	104
		**Gruul #6**	100% (166)	-	-	-	-	166
		**Gruul #7**	100% (87)	-	-	-	-	87
		**Gruul #8**	100% (101)	-	-	-	-	101
		**Mean**	100%	-	-	-	-	116.0
**F10-mC-mCe**	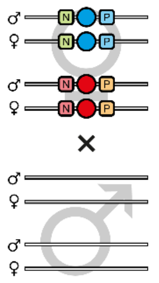	**Theoretical**	-	100%	-	-	-	-
		**Gruul #4**	-	100% (79)	-	-	-	79
		**Gruul #5**	-	100% (66)	-	-	-	66
		**Gruul #6**	-	100% (89)	-	-	-	89
		**Gruul #7**	-	100% (101)	-	-	-	101
		**Gruul #8**	-	100% (44)	-	-	-	44
		**Mean**	-	100%	-	-	-	75.8
**F10-mO-mVe**	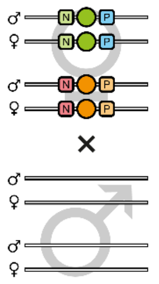	**Theoretical**	-	-	100%	-	-	-
		**Gruul #4**	-	-	100% (72)	-	-	72
		**Gruul #5**	-	-	100% (201)	-	-	201
		**Gruul #6**	-	-	100% (71)	-	-	71
		**Gruul #7**	-	-	100% (71)	-	-	71
		**Gruul #8**	-	-	100% (83)	-	-	83
		**Mean**	-	-	100%	-	-	99.6
**F10-mC-mVe**	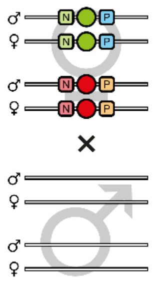	**Theoretical**	-	-	-	100%	-	-
		**Gruul #4**	-	-	-	100% (81)	-	81
		**Gruul #5**	-	-	-	100% (53)	-	53
		**Gruul #6**	-	-	-	100% (72)	-	72
		**Gruul #7**	-	-	-	100% (76)	-	76
		**Gruul #8**	-	-	-	100% (107)	-	107
		**Mean**	-	-	-	100%	-	77.8

The numbers in brackets and in the 'total' sub-column indicate the number of scored individuals. No significant differences between the arithmetic means and the theoretical Mendelian ratios were found. SD, standard deviation; n.s., not significant (*P*>0.05).

Biology Open • Supplementary information

Biology Open (2023): doi:10.1242/bio.060015: Supplementary information

Table S7. F9- and F10-associated control cross results for three repetitions of the Gruul #9 hybrid subline, which carries the AGOC and ACOS transgenes in close proximity on the same autosome.

Cross	Genotypes	Repetition	Progeny					
							other	total
F9-S	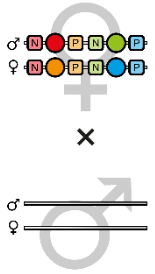	Theoretical	>25.0%	<25.0%	<25.0%	>25.0%	-	-
		Recombination Events	0×	1×	1×	0×	-	-
		Repetition 1	45.4% (119)	1.5% (4)	2.3% (6)	50.8% (133)	-	262
		Repetition 2	52.5% (134)	3.1% (8)	2.0% (5)	42.4% (108)	-	255
		Repetition 3	47.4% (102)	5.1% (11)	2.8% (6)	44.7% (96)	-	215
		Mean ± SD	48.4% ± 3.5%	3.2% ± 1.8%	2.4% ± 0.4%	46.0% ± 4.3%	-	244.0
		Significance	*P*<0.01	*P*<0.01	*P*<0.001	*P*<0.05	-	
F10-mO-mCe	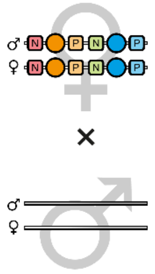	Theoretical	100%	-	-	-	-	-
		Repetition 1	100% (106)	-	-	-	-	106
		Repetition 2	100% (90)	-	-	-	-	90
		Repetition 3	100% (46)	-	-	-	-	46
		Mean	100%	-	-	-	-	80.7
F10-mC-mCe	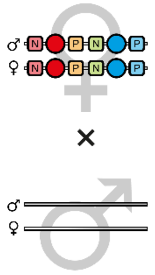	Theoretical	-	100%	-	-	-	-
		Repetition 1	-	100% (88)	-	-	-	88
		Repetition 2	-	100% (94)	-	-	-	94
		Repetition 3	-	100% (91)	-	-	-	91
		Mean	-	100%	-	-	-	91.0
F10-mO-mVe	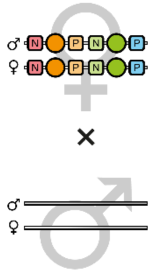	Theoretical	-	-	100%	-	-	-
		Repetition 1	-	-	100% (43)	-	-	43
		Repetition 2	-	-	100% (34)	-	-	34
		Repetition 3	-	-	100% (84)	-	-	84
		Mean	-	-	100%	-	-	53.7
F10-mC-mVe	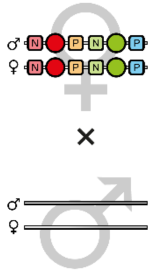	Theoretical	-	-	-	100%	-	-
		Repetition 1	-	-	-	100% (98)	-	98
		Repetition 2	-	-	-	100% (106)	-	106
		Repetition 3	-	-	-	100% (96)	-	96
		Mean	-	-	-	100%	-	100.0

The numbers in brackets and in the 'total' sub-column indicate the number of scored individuals. No significant differences between the arithmetic means and the theoretical Mendelian ratios were found. SD, standard deviation; n.s., not significant (*P*>0.05).

Biology Open • Supplementary information

Table S8. F9- and F10-associated control cross results for three repetitions of the Gruul #10 hybrid subline, which carries the AGOC transgene on the X allosome.

Cross	Genotypes	Repetition	Progeny					
							other	total
F9-S	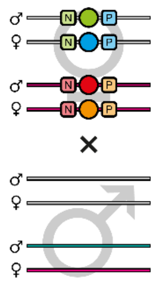	Theoretical	25.0%	25.0%	25.0%	25.0%	-	-
		Repetition 1	24.5% (12)	14.3% (7)	28.6% (14)	32.6% (16)	-	49
		Repetition 2	21.6% (24)	26.1% (29)	25.2% (28)	27.1% (30)	-	111
		Repetition 3	30.6% (30)	27.6% (27)	20.4% (20)	21.4% (21)	-	98
		Mean ± SD	25.6 ± 4.6%	22.7 ± 7.3%	24.7 ± 4.1%	27.0 ± 5.6%	-	86.0
		Significance	n.s.	n.s.	n.s.	n.s.	-	-
F10-mO-mCe-♀	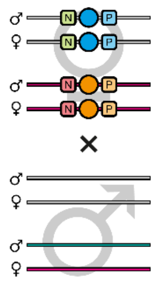	Theoretical	100%	-	-	-	-	-
		Repetition 1	100% (61)	-	-	-	-	61
		Repetition 2	100% (66)	-	-	-	-	66
		Repetition 3	100% (54)	-	-	-	-	54
		Mean	100%	-	-	-	-	60.3
F10-mC-mCe-♀	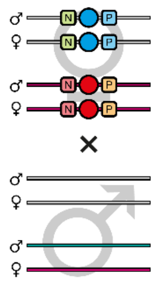	Theoretical	-	100%	-	-	-	-
		Repetition 1	-	100% (58)	-	-	-	58
		Repetition 2	-	100% (69)	-	-	-	69
		Repetition 3	-	100% (102)	-	-	-	102
		Mean	-	100%	-	-	-	76.3
F10-mO-mVe-♀	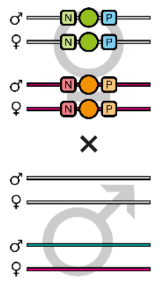	Theoretical	-	-	100%	-	-	-
		Repetition 1	-	-	100% (65)	-	-	65
		Repetition 2	-	-	100% (100)	-	-	100
		Repetition 3	-	-	100% (75)	-	-	75
		Mean	-	-	100%	-	-	80.0
F10-mC-mVe-♀	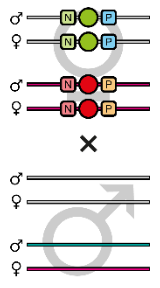	Theoretical	-	-	-	100%	-	-
		Repetition 1	-	-	-	100% (74)	-	74
		Repetition 2	-	-	-	100% (81)	-	81
		Repetition 3	-	-	-	100% (76)	-	76
		Mean	-	-	-	100%	-	77.0

Biology Open • Supplementary information

Cross	Genotypes	Repetition	Progeny							
									other	total
**F10-mO-mCe-♂**	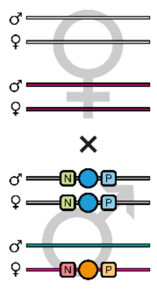	**Theoretical**	50.0%	-	50.0%	-	-	-	-	-
		**Repetition 1**	46.8% (36)	-	53.2% (41)	-	-	-	-	77
		**Repetition 2**	50.5% (46)	-	49.5% (45)	-	-	-	-	91
		**Repetition 3**	51.5% (50)	-	48.5% (47)	-	-	-	-	97
		**Mean ± SD**	49.6% ± 2.5%	-	50.4% ± 2.5%	-	-	-	-	88.3
		**Significance**	n.s.	-	n.s.	-	-	-	-	-
**F10-mC-mCe-♂**	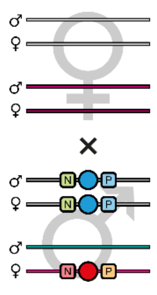	**Theoretical**	50.0%	-	-	50.0%	-	-	-	-
		**Repetition 1**	48.1% (51)	-	-	51.9% (55)	-	-	-	106
		**Repetition 2**	52.0% (64)	-	-	48.0% (59)	-	-	-	113
		**Repetition 3**	58.9% (33)	-	-	41.1% (23)	-	-	-	56
		**Mean ± SD**	53.0% ± 5.5%	-	-	47.0% ± 5.5%	-	-	-	91.7
		**Significance**	n.s.	-	-	n.s.	-	-	-	-
**F10-mO-mVe-♂**	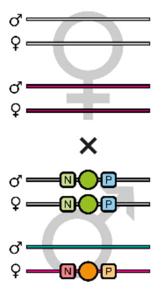	**Theoretical**	-	50.0%		-	50.0%	-	-	-
		**Repetition 1**	-	45.0% (49)		-	55.0% (60)	-	-	109
		**Repetition 2**	-	50.0% (25)		-	50.0% (25)	-	-	50
		**Repetition 3**	-	51.6% (49)		-	48.4% (46)	-	-	95
		**Mean ± SD**	-	48.9% ± 3.4%		-	51.1% ± 3.4%	-	-	84.7
		**Significance**	-	n.s.		-	n.s.	-	-	-
**F10-mC-mVe-♂**	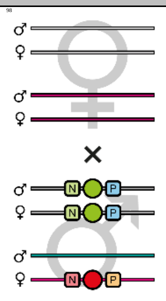	**Theoretical**	-	50.0%		-	-	50.0%	-	-
		**Repetition 1**	-	54.3% (38)		-	-	45.7% (32)	-	70
		**Repetition 2**	-	45.7% (37)		-	-	54.3% (44)	-	81
		**Repetition 3**	-	48.6% (51)		-	-	51.4% (54)	-	105
		**Mean ± SD**	-	49.5% ± 4.4%		-	-	50.5% ± 4.4%	-	85.3
		**Significance**	-	n.s.		-	-	n.s.	-	-

The numbers in brackets and in the 'total' sub-column indicate the number of scored individuals. No significant differences between the arithmetic means and the theoretical Mendelian ratios were found. SD, standard deviation; n.s., not significant (*P*>0.05).

Biology Open • Supplementary information

Table S9. Vector summary. The twenty vectors used/created in this study listed in order of their type. The numbers within the Source / molecular cloning column refer to the respective entry.

#	Vector	Type	Size	Resistance	Source / molecular cloning
01	pICE{HSP68'NLS-Cre}	previously described	5,709 bp	KanR	Vector described in (Strobl et al., 2018)
02	pAGOC{#P'#O(LA)-mEmerald}	previously described	6,852 bp	KanR	Vector described in (Strobl et al., 2018)
03	pGS[ACOS]	previously described	4,928 bp	KanR	Vector described in (Strobl et al., 2018)
04	pATub'piggyBac	previously described / helper	5,511 bp	AmpR	Vector described in (Strobl et al., 2018)
05	pGS[#O(MEM)-mRuby]	gene synthesis	3,170 bp	AmpR	Ordered from Thermo Fisher Scientific (796 bp *de novo* synthetized sequence inserted into the pMK-RQ backbone via SacI/KpnI)
06	pFIRE{HSP68'NLS-Cre]	Helper	5,700 bp	KanR	3×P3'mCherry cassette amplified from 02 with primer pair C10 and inserted into the AflII/AvrII sites of 01
07	pTC-'ATub-GEM-T Easy	#O library vector	4,363 bp	AmpR	'ATub amplified from cDNA with primer pair C6, A-tailed and inserted into pGEM-T Easy (copy & paste)
08	pTC-ATub'-GEM-T Easy	previously described / #P library	3,633 bp	AmpR	Vector described in (Strobl et al., 2018)
09	pTC-Ub'-GEM-T Easy	#P library	4,539 bp	AmpR	Ub' amplified from genomic DNA with primer pair C1, A-tailed and inserted into pGEM-T Easy (copy & paste)
10	pTC-EFA'-GEM-T Easy	#P library	6,480 bp	AmpR	EFA' amplified from genomic DNA with primer pair C2, A-tailed and inserted into pGEM-T Easy (copy & paste)
11	pTC-ATub'H2B-GEM-T Easy	previously described / #P'#O library	4,010 bp	AmpR	Vector described in (Strobl et al., 2018)
12	pACOS{#P'#O(LA)-mEmerald}	intermediate	6,870 bp	KanR	3×P3'mCerulean2 / 3×P3'mVenus cassette cut from 03 with AvrII/XhoI and inserted into the respective sites of 02
13	pACOS{#P'#O(MEM)-mRuby}	intermediate	6,873 bp	KanR	#O(MEM)-mRuby2 cassette cut from 05 with FseI/SbfI and inserted into the respective sites of 12
14	pACOS{#P'ATub-mRuby}	intermediate	8,163 bp	KanR	'ATub amplified from 07 with primer pair C6 and inserted into the FseI/NotI sites of 13
15	pACOS{ATub'H2B-mRuby}	previously described / transformation	7,730 bp	KanR	Vector described in (Strobl and Stelzer, 2021)s / ATub'H2B cut from 11 with AscI/NotI and inserted into the respective sites of 13
16	pACOS{ATub'#O(MEM)-mRuby}	transformation	7,421 bp	KanR	ATub' amplified from 08 with primer pair C4, digested with AscI/BsmBI and inserted into the respective sites of 13, which was digested with BtgZI
17	pACOS{Ub'#O(MEM)-mRuby}	transformation	8,325 bp	KanR	Ub' amplified from 09 with primer pair C5, digested with AscI/BsaI and inserted into the respective sites of 13, which was digested with BtgZI
18	pACOS{ATub'ATub-mRuby}	transformation	8,711 bp	KanR	ATub' amplified from 08 with primer pair C7, digested with AscI/BsmBI and inserted into the respective sites of 14, which was digested with BtgZI
19	pACOS{Ub'ATub-mRuby}	transformation	9,614 bp	KanR	Ub' amplified from 09 with primer pair C8, digested with AscI/BsaI and inserted into the respective sites of 14, which was digested with BtgZI
20	pACOS{ShEFA'ATub-mRuby}	transformation	9,689 bp	KanR	A shortened version of EFA' amplified with primer pair C9 from 10, digested with BsmBI and inserted into the respective sites of 14, which was digested with BtgZI

Biology Open • Supplementary information

Table S10. Molecular cloning and insertion junction sequencing primers.

Primer pair	#	Primer name	Primer sequence	Comment	Length	T_M*
C1	C1-1	TC Ub’ ExPCR FD	5’-GCGAAGAATACAACGAATCGTG-3’	-	22 bp	58.4°C
	C1-2	TC Ub’ ExPCR RV	5’-AAAGTCTTCACGAAGATCTGCATC-3’	-	24 bp	59.3°C
C2	C2-1	TC EFA’ ExPCR FD	5’-CACACTCCGTTATCTCGCGTTCA-3’	-	23 bp	62.4°C
	C2-2	TC EFA’ ExPCR RV	5’-CTACGTGGCCGATAACGACGATGTTG-3’	-	26 bp	66.4°C
C3	C3-1	TC ’ATub ExPCR FD	5’-ATGCGTGAATGTATCTCAGTTCATG-3'	NCBI Reference Sequence: XM_961399.3	25 bp	59.7°C
	C3-2	TC ’ATub ExPCR RV	5’-TTAATACTCTTCGCCACCTTCGC-3’	NCBI Reference Sequence: XM_961399.3	23 bp	60.6°C
C4	C4-1	ATub’ (ATGT) TrPCR FD	5’-AAATTT**GGCGCGCC**AGATGTCTATGTATCTCCCGTGAAAC-3’	overhang underlined; AscI site bold	40 bp	61.6°C
	C4-2	ATub’ (ATGT) TrPCR FD	5’-AAATTT**CGTCTC**AACATTTTGGTAGTTGAGTTTTACAAATTAC-3’	overhang underlined, BsmBI site bold	43 bp	58.2°C
C5	C5-1	Ub’ (ATGT) TrPCR FD	5’-AAATTT**GGCGCGCC**GCGAAGAATACAACGAATCGTG-3’	overhang underlined; AscI site bold	36 bp	58.4°C
	C5-2	Ub’ (ATGT) TrPCR RV	5’-AAATTT**GGTCTC**AACATCTGCAACGACACAAAAAAATTAC-3’	overhang underlined, BsaI site bold	39 bp	58.1°C
C6	C6-1	’ATub TrPCR FD	5’-AAATTT**GGCCGGCC**AAAATGCGTGAATGTATCTCAGTTCATG-3’	overhang underlined, FseI site bold	42 bp	59.7°C
	C6-2	’ATub TrPCR RV	5’-AAATTT**GCGGCCGC**ATACTCTTCGCCACCTTCGC-3’	overhang underlined, NotI site bold	34 bp	54.3°C
C7	C7-1	ATub’ (ATGC) TrPCR FD	5’-AAATTT**GGCGCGCC**AGATGTCTATGTATCTCCCGTGAAAC-3’	overhang underlined; AscI site bold	40 bp	61.6°C
	C7-2	ATub’ (ATGC) TrPCR FD	5’-AAATTT**CGTCTC**AGCATTTTGGTAGTTGAGTTTTACAAATTAC-3’	overhang underlined, BsmBI site bold	43 bp	58.2°C
C8	C8-1	Ub’ (ATGC) TrPCR FD	5’-AAATTT**GGCGCGCC**GCGAAGAATACAACGAATCGTG-3’	overhang underlined; AscI site bold	36 bp	58.4°C
	C8-2	Ub’ (ATGC) TrPCR RV	5’-AAATTT**GGTCTC**AGCATCTGCAACGACACAAAAAAATTAC-3’	overhang underlined, BsaI site bold	39 bp	58.1°C
C9	C9-1	ShEFA’ (ATGC) TrPCR FD	5’-AAATTT**CGTCTC**GCGCGCCGTGTCCATACTGGCAAGGAGCCC-3’	overhang underlined, BsmBI site bold	40 bp	66.0°C
	C9-2	ShEFA’ (ATGC) TrPCR RV	5’-AAATTT**CGTCTC**CGCATCTTGCTCCAACCTGCAAAACCACCC-3’	overhang underlined, BsmBI site bold	34 bp	68.0°C
C10	C10-1	3×P3’FP TrPCR FD	5’-AAATTT**CTTAAG**GTTCCCACAATGGTTAATTCGAG-3’	overhang underlined, AflII site bold	35 bp	58.9°C
	C10-2	3×P3’FP TrPCR RFV	5’-AAATTT**CCTAGG**TAAGATACATTGATGAGTTTGG-3’	overhang underlined, AvrII site bold	34 bp	52.8°C
I-3’	I-3’-1	AVOIAF 3’ InSeq FD	5’-AAAACTTGTTTAAACCCCGGG-3’	-	21 bp	55.9°C
	I-3’-2	AVOIAF 3’ InSeq RV	5’-TAAATAAATTGTTTGTTGAATTTATTATTAGTATGTAAGTG-3’	-	41 bp	60.4°C
I-5’	I-5’-1	AVOIAF 5’ InSeq FD	5’-TCTTGTTATAGATATCAGTTTAAACCCTAGG-3’	-	31 bp	61.6°C
	I-5’-2	AVOIAF 5’ InSeq RV	5’-GCGTCATTTTGACTCACGCGG-3’	-	21 bp	61.8°C
-	I-3’-Seq	AVOIAF 3’ InSeq Sequencing	5’-TTATTATTAGTATGTAAGTGTAAATATAATAAAAC-3’	-	35 bp	56.6°C
	I-5’-Seq	AVOIAF 5’ InSeq Sequencing	5’-TTTGACTCACGCGGTCGTTATAG-3’	-	25 bp	60.6°C
I1	I1-1	ACOS{ATub’#O(MEM)-mRuby} #1 3’ ConPCR FD	5’-ATTTAAAAGTAATTTATACAGCGTGGTCC-3’	-	29 bp	59.6°C
	I1-2	ACOS{ATub’#O(MEM)-mRuby} #1 3’ ConPCR RV	5’-TAAATAAATTGTTTGTTGAATTTATTATTAGTATGTAAGTG-3’	-	41 bp	60.4°C
I2	I2-1	ACOS{ATub’#O(MEM)-mRuby} #2 5’ ConPCR FD	5’-GCGTCATTTTGACTCACGCGG-3’	-	21 bp	61.8°C
	I2-2	ACOS{ATub’#O(MEM)-mRuby} #2 5’ ConPCR RV	5’-GGATACGATCAAATTACCAAAGCAG-3’	-	25 bp	59.7°C
I3	I3-1	ACOS{ATub’H2B-mRuby} #1 3’ ConPCR FD	5’-AGGTTCAACAGATGATTAACTTATTTACAAC-3’	-	31 bp	60.2°C
	I3-2	ACOS{ATub’H2B-mRuby} #1 3’ ConPCR RV	5’-TAAATAAATTGTTTGTTGAATTTATTATTAGTATGTAAGTG-3’	-	41 bp	60.4°C
I4	I4-1	ACOS{ATub’H2B-mRuby} #2 3’ ConPCR FD	5’-GCGTCATTTTGACTCACGCGG-3’	-	21 bp	61.8°C
	I4-2	ACOS{ATub’H2B-mRuby} #2 3’ ConPCR RV	5’-CTTGGCGAGGTGGACGCCC-3’	-	19 bp	65.3°C
I5	I5-1	ACOS{ATub’ATub-mRuby} #1 5’ ConPCR FD	5'-GCGTCATTTTGACTCACGCGG-3'	-	21 bp	61.8°C
	I5-2	ACOS{ATub’ATub-mRuby} #1 5’ ConPCR RV	5'-CACTCCCAACATGAGCATTCATTTG-3'	-	25 bp	61.3°C
I6	I6-1	ACOS{ATub’ATub-mRuby} #2 5’ ConPCR FD	5'-GCGTCATTTTGACTCACGCGG-3'	-	21 bp	61.8°C
	I6-2	ACOS{ATub’ATub-mRuby} #2 5’ ConPCR RV	5'-TTGATTTAATGGTGGGAATACTATTTTTTCG-3'	-	31 bp	60.2°C
I7	I7-1	ACOS{Ub’ATub-mRuby} #1 5’ ConPCR FD	5'-GCGTCATTTTGACTCACGCGG-3'	-	21 bp	61.8°C
	I7-2	ACOS{Ub’ATub-mRuby} #1 5’ ConPCR RV	5'-TAGCGACAATAAAAACCAGACTTCAC-3'	-	26 bp	60.1°C
I8	I8-1	ACOS{Ub’ATub-mRuby} #2 5’ ConPCR FD	5'-GCGTCATTTTGACTCACGCGG-3'	-	21 bp	61.8°C
	I8-2	ACOS{Ub’ATub-mRuby} #2 5’ ConPCR RV	5'-TTATTATGTCTACCCAAGTGCAATGG-3'	-	26 bp	60.1°C
I9	I9-1	ACOS{ShEFA’ATub-mRuby} #2 3’ ConPCR FD	5'-CTGTCCGGATTGATGCTCAAAG-3'	-	22 bp	60.3°C
	I9-2	ACOS{ShEFA’ATub-mRuby} #2 3’ ConPCR RV	5'-TAAATAAATTGTTTGTTGAATTTATTATTAGTATGTAAGTG-3'	-	41 bp	60.4°C

* T_M was calculated with Geneious (without considering the overhang, if applicable)

Biology Open • Supplementary information

Table S11. Imaging Metadata.

Dataset (DS)	**DS0001**	**DS0002**	**DS0003**
Species		*Tribolium castaneum* (Herbst)<br>Arthropoda → Insecta → Coleoptera → Tenebrionidae	
Line / Subline		ACOS{Ub'ATub-mRuby} #2	
Genotype		single insertion<br>(mCe/mCe) homozygous	
Imaging Culture		~200-500 adults, less than 2 months old	
Imaging Culture Medium		full grain wheat flour (SP061036, Demeter, Darmstadt, Germany)<br>supplemented with 5% (wt/wt) inactive dry yeast<br>(62-106, Flystuff, San Diego, CA, USA)	
Imaging Culture Rearing Conditions		12:00 h light / 12:00 h darkness at 32°C and 70% relative humidity<br>(DR-36VL, Percival Scientific, Perry, IA, USA)	
Embryo Collection		imaging cultures were given 01:00 h at room temperature (23±1°C)<br>in egg laying medium (405 fine wheat flour (SP061006, Demeter, Darmstadt, Germany)<br>supplemented with 5% (wt/wt) inactive dry yeast (62-106, Flystuff, San Diego, CA, USA) to lay embryos	
Pre-Imaging Incubation		incubation for ~14:00 h at 20°C,<br>embryo mounting for ~01:00 h at room temperature (23±1°C)	
LSFM Implementation		sample chamber-based digital scanned laser light sheet fluorescence microscope (DSLM)<br>(Keller and Stelzer 2010, *Cold Spring Harbor Protocols* (doi.org/10.1101/pdb.top78))	
Laser Lines		561 nm / 25 mW DPSSL<br>(Cobolt Jive CW 561, Omicron Laserprodukte GmbH, Rodgau-Dudenhofen, Germany)	
Excitation Objective		2.5× NA 0.06 EC Epiplan-Neofluar objective (422320-9900-000**,** Carl Zeiss, Göttingen, Germany)	
Emission Objective		10× NA 0.3 W N-Achroplan objective (420947-9900-000, Carl Zeiss, Göttingen, Germany)	
Emission Filters		607/70 single-band bandpass filter<br>(FF01-607/70-25, Semrock/AHF Analysentechnik AG, Tübingen, Germany)	
Camera		high-resolution CCD (Clara, Andor, Belfast, United Kingdom), 14 bit, 1040×1392 pixel (pitch 6.45 µm)	
Dataset File Type		TIFF, 16-bit grayscale (planes saved as Z stacks in ZIP-compressed container files, indicated as PL(ZS))	
Pre-Mounting Preparation		dechorionation by washing ~30 s in a 1:9 mixture of<br>~10% (vol/vol) sodium hypochlorite (425044-250ML, Sigma Aldrich, Taufkirchen, Germany)<br>and autoclaved tap water	
Mounting Method		cobweb holder mounting method (embryos are attached to a thin agarose film spanning a slotted hole)<br>(Strobl *et al*. 2017, *Journal of Visualized Experiments* (doi.org/10.3791/55629))	
Mounting Agarose		1% (wt/vol) low-melt agarose (6351.2, Carl Roth, Karlsruhe, Germany)<br>in autoclaved tap water	
Imaging Buffer		autoclaved tap water	
Imaging Temperature		room temperature (23°C ± 1°C)	

Dataset (DS)	**DS0001**	**DS0002**	**DS0003**
Embryogenic Events	blastoderm formation (partially)	blastoderm formation (partially)	blastoderm formation (partially)
Views	unknown	unknown	unknown
Retrieval	developed to healthy adult, produced fertile progeny	developed to healthy adult, produced fertile progeny	developed to healthy adult, produced fertile progeny
Dataset Size	2.47 Gigabyte	2.65 Gigabyte	2.51 Gigabyte
Dataset Access	doi.org/10.5281/zenodo.7564542	doi.org/10.5281/zenodo.7564542	doi.org/10.5281/zenodo.7564542
Figures	3A	-	-
Supplementary Figures	4	-	-
Supplementary Movies	1	1	1
Comment	-	-	chorion fragments remained on top of the vitelline membrane
Time Points (TP)	**41 (TP0001-TP0041)**	**41 (TP0001-TP0041)**	**41 (TP0001-TP0041)**
TP Interval	00:06 h	00:06 h	00:06 h
Total Time ((TP-1)×TP Interval)	04:00 h	04:00 h	04:00 h
Directions (DR)	**1 (DR0001)**	**1 (DR0001)**	**1 (DR0001)**
DR Orientations	0°	0°	0°
Channels (CH)	**1 (CH0001)**	**1 (CH0001)**	**1 (CH0001)**
CH0001 Excitation	561 nm	561 nm	561 nm
CH0001 Laser Power	135 µW (close to the embryo)	135 µW (close to the embryo)	135 µW (close to the embryo)
CH0001 Exposure Time	100 ms	100 ms	100 ms
CH0001 Emission Filter	607/70 single-band bandpass filter	607/70 single-band bandpass filter	607/70 single-band bandpass filter
CH0001 Theoretical Lateral / Axial Resolution	~1,000 nm / ~3,700 nm	~1,000 nm / ~3,700 nm	~1,000 nm / ~3,700 nm
Planes (PL)	**150 (PL0001-PL0150)**	**150 (PL0001-PL0150)**	**150 (PL0001-PL0100)**
Z Spacing	2.58 µm	2.58 µm	2.58 µm
Z Distance (PL×Z Spacing)	387.0 µm	387.0 µm	387.0 µm
X-Dimensions (XD)	**600 pixels**	**600 pixels**	**600 pixels**
X Spacing	0.645 µm	0.645 µm	0.645 µm
X Length (XD×X Spacing)	387.0 µm	387.0 µm	387.0 µm
Y-Dimensions (YD)	**1000 pixels**	**1100 pixels**	**1000 pixels**
Y Spacing	0.645 µm	0.645 µm	0.645 µm
Y Length (YD×Y Spacing)	645.0 µm	709.5 µm	645.0 µm

Dataset (DS)	**DS0004**	**DS0005**	**DS0006**
Species	*Tribolium castaneum* (Herbst)<br>Arthropoda → Insecta → Coleoptera → Tenebrionidae		
Line / Subline	ACOS{Ub'ATub-mRuby} #2		
Genotype	single insertion<br>(mVe/mVe) homozygous		
Imaging Culture	~200-500 adults, less than 2 months old		
Imaging Culture Medium	full grain wheat flour (SP061036, Demeter, Darmstadt, Germany)<br>supplemented with 5% (wt/wt) inactive dry yeast<br>(62-106, Flystuff, San Diego, CA, USA)		
Imaging Culture Rearing Conditions	12:00 h light / 12:00 h darkness at 32°C and 70% relative humidity<br>(DR-36VL, Percival Scientific, Perry, IA, USA)		
Embryo Collection	imaging cultures were given 01:00 h at room temperature (23±1°C)<br>in egg laying medium (405 fine wheat flour (SP061006, Demeter, Darmstadt, Germany)<br>supplemented with 5% (wt/wt) inactive dry yeast (62-106, Flystuff, San Diego, CA, USA)) to lay embryos		
Pre-Imaging Incubation	incubation for ~14:00 h at 20°C,<br>embryo mounting for ~01:00 h at room temperature (23±1°C)		
LSFM Implementation	sample chamber-based digital scanned laser light sheet fluorescence microscope (DSLM)<br>(Keller and Stelzer 2010, *Cold Spring Harbor Protocols* (doi.org/10.1101/pdb.top78))		
Laser Lines	561 nm / 25 mW DPSSL<br>(Cobolt Jive CW 561, Omicron Laserprodukte GmbH, Rodgau-Dudenhofen, Germany)		
Excitation Objective	2.5× NA 0.06 EC Epiplan-Neofluar objective (422320-9900-000**,** Carl Zeiss, Göttingen, Germany)		
Emission Objective	10× NA 0.3 W N-Achroplan objective (420947-9900-000, Carl Zeiss, Göttingen, Germany)		
Emission Filters	607/70 single-band bandpass filter<br>(FF01-607/70-25, Semrock/AHF Analysentechnik AG, Tübingen, Germany)		
Camera	high-resolution CCD (Clara, Andor, Belfast, United Kingdom), 14 bit, 1040×1392 pixel (pitch 6.45 µm)		
Dataset File Type	TIFF, 16-bit grayscale (planes saved as Z stacks in ZIP-compressed container files, indicated as PL(ZS))		
Pre-Mounting Preparation	dechorionation by washing ~30 s in a 1:9 mixture of<br>~10% (vol/vol) sodium hypochlorite (425044-250ML, Sigma Aldrich, Taufkirchen, Germany)<br>and autoclaved tap water		
Mounting Method	cobweb holder mounting method (embryos are attached to a thin agarose film spanning a slotted hole)<br>(Strobl *et al*. 2017, *Journal of Visualized Experiments* (doi.org/10.3791/55629))		
Mounting Agarose	1% (wt/vol) low-melt agarose (6351.2, Carl Roth, Karlsruhe, Germany)<br>in autoclaved tap water		
Imaging Buffer	autoclaved tap water		
Imaging Temperature	room temperature (23°C ± 1°C)		

Biology Open • Supplementary information

Dataset (DS)	**DS0004**	**DS0005**	**DS0006**
Embryonic Events	blastoderm formation (partially)	blastoderm formation (partially)	blastoderm formation (partially)
Views	unknown	unknown	unknown
Retrieval	developed to healthy adult, produced fertile progeny	developed to healthy adult, produced fertile progeny	developed to healthy adult, produced fertile progeny
Dataset Size	2.56 Gigabyte	2.46 Gigabyte	2.62 Gigabyte
Dataset Access	doi.org/10.5281/zenodo.7564542	doi.org/10.5281/zenodo.7564542	doi.org/10.5281/zenodo.7564542
Figures	-	-	-
Supplementary Figures	4	-	-
Supplementary Movies	1	1	1
Comment	chorion fragments remained on top of the vitelline membrane	chorion fragments remained on top of the vitelline membrane	chorion fragments remained on top of the vitelline membrane
Time Points (TP)	**41 (TP0001-TP0041)**	**41 (TP0001-TP0041)**	**41 (TP0001-TP0041)**
TP Interval	00:06 h	00:06 h	00:06 h
Total Time ((TP-1)×TP Interval)	04:00 h	04:00 h	04:00 h
Directions (DR)	**1 (DR0001)**	**1 (DR0001)**	**1 (DR0001)**
DR Orientations	0°	0°	0°
Channels (CH)	**1 (CH0001)**	**1 (CH0001)**	**1 (CH0001)**
CH0001 Excitation	561 nm	561 nm	561 nm
CH0001 Laser Power	135 µW (close to the embryo)	135 µW (close to the embryo)	135 µW (close to the embryo)
CH0001 Exposure Time	100 ms	100 ms	100 ms
CH0001 Emission Filter	607/70 single-band bandpass filter	607/70 single-band bandpass filter	607/70 single-band bandpass filter
CH0001 Theoretical Lateral / Axial Resolution	~1,000 nm / ~3,700 nm	~1,000 nm / ~3,700 nm	~1,000 nm / ~3,700 nm
Planes (PL)	**150 (PL0001-PL0150)**	**150 (PL0001-PL0150)**	**150 (PL0001-PL0100)**
Z Spacing	2.58 µm	2.58 µm	2.58 µm
Z Distance (PL×Z Spacing)	387.0 µm	387.0 µm	387.0 µm
X-Dimensions (XD)	**600 pixels**	**600 pixels**	**600 pixels**
X Spacing	0.645 µm	0.645 µm	0.645 µm
X Length (XD×X Spacing)	387.0 µm	387.0 µm	387.0 µm
Y-Dimensions (YD)	**1000 pixels**	**1000 pixels**	**1100 pixels**
Y Spacing	0.645 µm	0.645 µm	0.645 µm
Y Length (YD×Y Spacing)	645.0 µm	645.0 µm	709.5 µm

Dataset (DS)	**DS0007**	**DS0008**
Species	*Tribolium castaneum* (Herbst)<br>Arthropoda → Insecta → Coleoptera → Tenebrionidae	
Line / Subline	Gruul #8	
Genotype	single insertions<br>(mO/mO; mCe/mCe) double homozygous	single insertions<br>(mC/mC; mCe/mCe) double homozygous
Imaging Culture	~200-500 adults, less than 2 months old	
Imaging Culture Medium	full grain wheat flour (SP061036, Demeter, Darmstadt, Germany)<br>supplemented with 5% (wt/wt) inactive dry yeast<br>(62-106, Flystuff, San Diego, CA, USA)	
Imaging Culture Rearing Conditions	12:00 h light / 12:00 h darkness at 32°C and 70% relative humidity<br>(DR-36VL, Percival Scientific, Perry, IA, USA)	
Embryo Collection	imaging cultures were given 01:00 h at room temperature (23±1°C)<br>in egg laying medium (405 fine wheat flour (SP061006, Demeter, Darmstadt, Germany)<br>supplemented with 5% (wt/wt) inactive dry yeast (62-106, Flystuff, San Diego, CA, USA) to lay embryos	
Pre-Imaging Incubation	incubation for ~14:00 h at 20°C,<br>embryo mounting for ~01:00 h at room temperature (23±1°C)	
LSFM Implementation	sample chamber-based digital scanned laser light sheet fluorescence microscope (DSLM)<br>(Keller and Stelzer 2010, *Cold Spring Harbor Protocols* (doi.org/10.1101/pdb.top78))	
Laser Lines	561 nm / 25 mW DPSSL<br>(Cobolt Jive CW 561, Omicron Laserprodukte GmbH, Rodgau-Dudenhofen, Germany)	
Excitation Objective	2.5× NA 0.06 EC Epiplan-Neofluar objective (422320-9900-000, Carl Zeiss, Göttingen, Germany)	
Emission Objective	10× NA 0.3 W N-Achroplan objective (420947-9900-000, Carl Zeiss, Göttingen, Germany)	
Emission Filters	607/70 single-band bandpass filter<br>(FF01-607/70-25, Semrock/AHF Analysentechnik AG, Tübingen, Germany)	
Camera	high-resolution CCD (Clara, Andor, Belfast, United Kingdom), 14 bit, 1040×1392 pixel (pitch 6.45 µm)	
Dataset File Type	TIFF, 16-bit grayscale (planes saved as Z stacks in ZIP-compressed container files, indicated as PL(ZS))	
Pre-Mounting Preparation	dechorionation by washing ~30 s in a 1:9 mixture of<br>~10% (vol/vol) sodium hypochlorite (425044-250ML, Sigma Aldrich, Taufkirchen, Germany)<br>and autoclaved tap water	
Mounting Method	cobweb holder mounting method (embryos are attached to a thin agarose film spanning a slotted hole)<br>(Strobl *et al*. 2017, *Journal of Visualized Experiments* (doi.org/10.3791/55629))	
Mounting Agarose	1% (wt/vol) low-melt agarose (6351.2, Carl Roth, Karlsruhe, Germany)<br>in autoclaved tap water	
Imaging Buffer	autoclaved tap water	
Imaging Temperature	room temperature (23°C ± 1°C)	

Biology Open • Supplementary information

Dataset (DS)	**DS0007**	**DS0008**
Embryogenic Events	blastoderm formation (partially)	blastoderm formation (partially)
Views	unknown	unknown
Retrieval	developed to healthy adult, produced fertile progeny	developed to healthy adult, produced fertile progeny
Dataset Size	4.70 Gigabyte (TIFF)	4.90 Gigabyte (TIFF)
Dataset Access	doi.org/10.5281/zenodo.7564542	doi.org/10.5281/zenodo.7564542
Figures	7A, 7B, 7C	7C
Supplementary Figures	-	-
Supplementary Movies	2	2
Comment	chorion fragments remained on top of the vitelline membrane	chorion fragments remained on top of the vitelline membrane
Time Points (TP)	**41 (TP0001-TP0041)**	**41 (TP0001-TP0041)**
TP Interval	00:06 h	00:06 h
Total Time ((TP-1)×TP Interval)	04:00 h	04:00 h
Directions (DR)	**1 (DR0001)**	**1 (DR0001)**
DR Orientations	0°	0°
Channels (CH)	**2 (CH0001-CH0002)**	**2 (CH0001-CH0002)**
CH0001 Excitation	488 nm	488 nm
CH0001 Laser Power	135 µW (close to the embryo)	135 µW (close to the embryo)
CH0001 Exposure Time	100 ms	100 ms
CH0001 Emission Filter	525/50 single-band bandpass filter	525/50 single-band bandpass filter
CH0001 Theoretical Lateral / Axial Resolution	~850 nm / ~3,200 nm	~850 nm / ~3,200 nm
CH0002 Excitation	561 nm	561 nm
CH0002 Laser Power	135 µW (close to the embryo)	135 µW (close to the embryo)
CH0002 Exposure Time	100 ms	100 ms
CH0002 Emission Filter	607/70 single-band bandpass filter	607/70 single-band bandpass filter
CH0002 Theoretical Lateral / Axial Resolution	~1,000 nm / ~3,700 nm	~1,000 nm / ~3,700 nm
Planes (PL)	**150 (PL0001-PL0150)**	**150 (PL0001-PL0150)**
Z Spacing	2.58 µm	2.58 µm
Z Distance (PL×Z Spacing)	387.0 µm	387.0 µm
X-Dimensions (XD)	**600 pixels**	**600 pixels**
X Spacing	0.645 µm	0.645 µm
X Length (XD×X Spacing)	387.0 µm	387.0 µm
Y-Dimensions (YD)	**1000 pixels**	**1000 pixels**
Y Spacing	0.645 µm	0.645 µm
Y Length (YD×Y Spacing)	645.0 µm	645.0 µm

Biology Open • Supplementary information

Dataset (DS)	**DS0009**	**DS0010**
Species	*Tribolium castaneum* (Herbst)<br>Arthropoda → Insecta → Coleoptera → Tenebrionidae	
Line / Subline	Gruul #8	
Genotype	single insertions<br>(mO/mO; mVe/mVe) double homozygous	single insertions<br>(mC/mC; mVe/mVe) double homozygous
Imaging Culture	~200-500 adults, less than 2 months old	
Imaging Culture Medium	full grain wheat flour (SP061036, Demeter, Darmstadt, Germany)<br>supplemented with 5% (wt/wt) inactive dry yeast<br>(62-106, Flystuff, San Diego, CA, USA)	
Imaging Culture Rearing Conditions	12:00 h light / 12:00 h darkness at 32°C and 70% relative humidity<br>(DR-36VL, Percival Scientific, Perry, IA, USA)	
Embryo Collection	imaging cultures were given 01:00 h at room temperature (23±1°C)<br>in egg laying medium (405 fine wheat flour (SP061006, Demeter, Darmstadt, Germany)<br>supplemented with 5% (wt/wt) inactive dry yeast (62-106, Flystuff, San Diego, CA, USA) to lay embryos	
Pre-Imaging Incubation	incubation for ~14:00 h at 20°C,<br>embryo mounting for ~01:00 h at room temperature (23±1°C)	
LSFM Implementation	sample chamber-based digital scanned laser light sheet fluorescence microscope (DSLM)<br>(Keller and Stelzer 2010, *Cold Spring Harbor Protocols* (doi.org/10.1101/pdb.top78))	
Laser Lines	561 nm / 25 mW DPSSL<br>(Cobolt Jive CW 561, Omicron Laserprodukte GmbH, Rodgau-Dudenhofen, Germany)	
Excitation Objective	2.5× NA 0.06 EC Epiplan-Neofluar objective (422320-9900-000**,** Carl Zeiss, Göttingen, Germany)	
Emission Objective	10× NA 0.3 W N-Achroplan objective (420947-9900-000, Carl Zeiss, Göttingen, Germany)	
Emission Filters	607/70 single-band bandpass filter<br>(FF01-607/70-25, Semrock/AHF Analysentechnik AG, Tübingen, Germany)	
Camera	high-resolution CCD (Clara, Andor, Belfast, United Kingdom), 14 bit, 1040×1392 pixel (pitch 6.45 µm)	
Dataset File Type	TIFF, 16-bit grayscale (planes saved as Z stacks in ZIP-compressed container files, indicated as PL(ZS))	
Dechorionation	~30 s in 10% (vol/vol) sodium hypochlorite (425044-250ML, Sigma Adlrich, Taufkirchen, Germany)<br>in autoclaved tap water	
Mounting Method	cobweb holder mounting method (embryos are attached to a thin agarose film spanning a slotted hole)<br>(Strobl *et al*. 2017, *Journal of Visualized Experiments* (doi.org/10.3791/55629))	
Mounting Agarose	1% (wt/vol) low-melt agarose (6351.2, Carl Roth, Karlsruhe, Germany)<br>in autoclaved tap water	
Imaging Buffer	autoclaved tap water	
Imaging Temperature	room temperature (23°C ± 1°C)	

Biology Open • Supplementary information

Dataset (DS)	DS0009	DS0010
Embryogenic Events	blastoderm formation (partially)	blastoderm formation (partially)
Views	unknown	unknown
Retrieval	developed to healthy adult, produced fertile progeny	developed to healthy adult, produced fertile progeny
Dataset Size	4.86 Gigabyte (TIFF)	4.85 Gigabyte (TIFF)
Dataset Access	doi.org/10.5281/zenodo.7564542	doi.org/10.5281/zenodo.7564542
Figures	7C	7C
Supplementary Figures	-	-
Supplementary Movies	2	2
Comment	chorion fragments remained on top of the vitelline membrane	chorion fragments remained on top of the vitelline membrane
Time Points (TP)	**41 (TP0001-TP0041)**	**41 (TP0001-TP0041)**
TP Interval	00:06 h	00:06 h
Total Time ((TP-1)×TP Interval)	04:00 h	04:00 h
Directions (DR)	**1 (DR0001)**	**1 (DR0001)**
DR Orientations	0°	0°
Channels (CH)	**2 (CH0001-CH0002)**	**2 (CH0001-CH0002)**
CH0001 Excitation	488 nm	488 nm
CH0001 Laser Power	135 µW (close to the embryo)	135 µW (close to the embryo)
CH0001 Exposure Time	100 ms	100 ms
CH0001 Emission Filter	525/50 single-band bandpass filter	525/50 single-band bandpass filter
CH0001 Theoretical Lateral / Axial Resolution	~850 nm / ~3,200 nm	~850 nm / ~3,200 nm
CH0002 Excitation	561 nm	561 nm
CH0002 Laser Power	135 µW (close to the embryo)	135 µW (close to the embryo)
CH0002 Exposure Time	100 ms	100 ms
CH0002 Emission Filter	607/70 single-band bandpass filter	607/70 single-band bandpass filter
CH0002 Theoretical Lateral / Axial Resolution	~1,000 nm / ~3,700 nm	~1,000 nm / ~3,700 nm
Planes (PL)	**150 (PL0001-PL0150)**	**150 (PL0001-PL0150)**
Z Spacing	2.58 µm	2.58 µm
Z Distance (PL×Z Spacing)	387.0 µm	387.0 µm
X-Dimensions (XD)	**600 pixels**	**600 pixels**
X Length (XD×X Spacing)	387.0 µm	387.0 µm
Y-Dimensions (YD)	**1000 pixels**	**1000 pixels**
Y Spacing	0.645 µm	0.645 µm
Y Length (YD×Y Spacing)	645.0 µm	645.0 µm

File S1. All vector sequences are provided as GenBank (.gb) and Geneious (.geneious) files compressed into a single zipped folder (.zip).

Click here to download File S1

File S2. Raw scores tables for all mating procedure results ordered by transgenic sublines/hybrid sublines.

Click here to download File S2

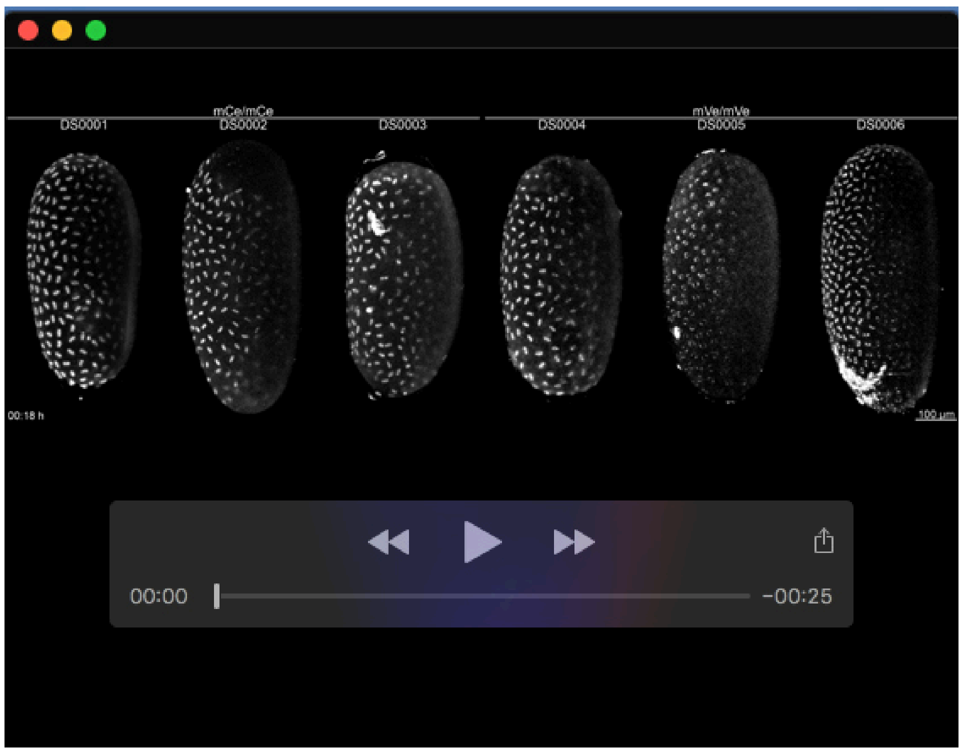

Movie 1. Fluorescence live imaging of homozygous ACOS{Ub'ATub-mRuby} #2 embryos during blastoderm formation using light sheet fluorescence microscopy. This subline expresses mRuby2-labeled *tubulin alpha 1-like protein* under control of the *polyubiquitin* promoter. Comparison time series of a (mCe/mCe) and a (mVe/mVe) embryo proceeding through the 10th, 11th, and 12th synchronous division waves. Both flavors show similar fluorescence patterns.

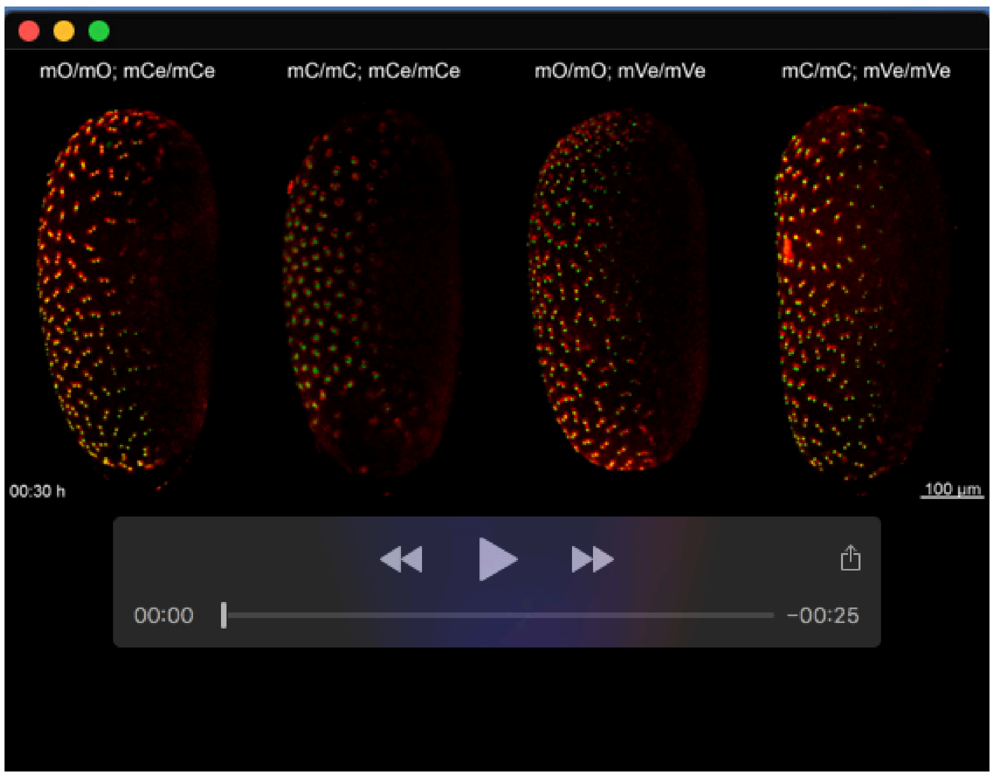

Movie 2. Fluorescence live imaging of double homozygous Gruul #8 embryos during blastoderm formation in two channels using light sheet fluorescence microscopy. This hybrid subline expresses mEmerald-labeled histone H2B under control of the *tubulin alpha 1-like protein* promoter and mRuby2-labeled *tubulin alpha 1-like protein* under control of a shortened version of the *elongation factor 1-alpha* promoter. Comparison of time series of one embryo from each flavor proceeding through the 10th, 11th, and 12th synchronous division waves. All flavors show similar fluorescence patterns.

Biology Open • Supplementary information
